# Fruit and Vegetable Supplemented-Diet Ameliorates Dextran Sodium Sulfate (DSS)-Induced Colitis by Modulating Host Transcriptome and Gut Metagenome Response

**DOI:** 10.3390/nu18060937

**Published:** 2026-03-16

**Authors:** Gloria Solano-Aguilar, Sukla Lakshman, Celine Chen, Ethiopia Beshah, Aleksey Molokin, Bryan Vinyard, Harry D. Dawson, Monica Santin-Duran, Gonzalo Bruna, Allen Smith, Joseph F. Urban

**Affiliations:** 1 Diet Genomics and Immunology Laboratory, Beltsville Human Nutrition Research Center, Agricultural Research Service, United States Department of Agriculture, NorthEast Area, Beltsville, MD 20705, USAethiopia.beshah@usda.gov (E.B.);; 2 Environmental Microbial and Food Safety Laboratory, Beltsville Agricultural Research Center, Agricultural Research Service, United States Department of Agriculture, NorthEast Area, Beltsville, MD 20705, USA; aleksey.molokin@usda.gov (A.M.); monica.santin-duran@usda.gov (M.S.-D.); 3 Statistics and Bioinformatics Group, Agricultural Research Service, United States Department of Agriculture, NorthEast Area, Beltsville, MD 20705, USA; 4 Seysa, 28047 Madrid, Spain; gbruna@seysa.com

**Keywords:** fruit and vegetable, DSS, colitis, metagenome, RNA-sequencing, PC mucosa

## Abstract

**Background/Objectives**: Dietary intake of fruits and vegetables (FVs) has been inversely associated with a lower risk of ulcerative colitis. Using a pig model, we evaluated the effect of FV supplementation on dextran sulfate sodium (DSS)-induced colitis. **Methods**: Six-week-old pigs were fed a grower diet (negative control), grower diet + 4% DSS (positive control), half-FV diet + DSS, or full-FV diet + DSS. FV levels matched half or full daily recommendations from the Dietary Guidelines for Americans (DGA). Clinical signs were monitored; proximal colon contents (PCs) and mucosa (PCM) were analyzed for metagenome, transcriptome and histopathology. **Results**: Full-FV pigs showed no diarrhea, less fecal occult blood (FOB), crypt hyperplasia, but no changes in gene expression or microbiome diversity (*p* < 0.05). Half-FV pigs had increased FOB, differentially expressed genes (DEGs) linked to tissue remodeling, crypt/goblet cell hyperplasia and two cases of diarrhea (*p* < 0.05). DSS controls showed reduced immune-related DEGs, altered microbiome, PCM erosion, FOB, and persistent diarrhea in one pig (*p* < 0.05). **Conclusions**: A three-week full-FV diet conferred protection against DSS-induced colitis, with a dose-dependent protection of intestinal tissue and gut metagenome under inflammatory challenge.

## 1. Introduction

Inflammatory bowel disease (IBD) refers to chronic inflammatory disorders that affect the gastrointestinal tract. There are two clinical forms of IBD: Crohn’s disease (CD), which can affect any part of the gastrointestinal tract, often in a noncontiguous manner, and ulcerative colitis, (UC) where pathology is restricted to the colonic mucosa [1]. UC affects significant numbers of people worldwide [2], including 1% of the US adult population [3]. Multiple environmental factors, including lifestyle, genetics and diet, affect the risk of developing UC; however, the mechanisms responsible for disease variability in UC cannot be fully explained by known risk factors [4,5]. Dysfunction of the intestinal mucosa due to abnormal signaling caused by pathogens, excessive leakage of bacterial antigens, or an altered immune response resulting from microbiome dysbiosis has been shown to contribute to an increased inflammatory response. This progressive inflammation degrades the intestinal epithelium, damages the mucosal barrier, and leads to ulcerations and severe bleeding [1,6,7]. Despite an increase in therapeutic options, some patients do not fully respond to treatment [8], and remission occurs in only 30–60% of cases [2]. Treatments such as antibiotics [9], fecal transplantation [4], and probiotics [10] have shown inconsistent benefits in managing IBD.

Given the role of dietary compounds and phytochemicals in modulating gut bacterial abundance and their interaction with human metabolism [11,12], there is growing interest in understanding how diet may affect gut inflammation. Variations in dietary habits, host genetics, gut microbiome composition, and their interactions have been proposed as risk factors for IBD [13,14]. Consumption of diets with increased intake of fruits and vegetables (FVs) [15], high fiber intake [16,17], and reduced consumption of processed meats and refined carbohydrates has been inversely associated with a lower risk of UC [18]. Differences in dietary components alter the composition of gut microbiota, resulting in different metabolite profiles that affect host physiology [16,19,20,21]. However, inconsistencies in IBD microbiome characterization have been attributed to several factors, including differences in microbiome sampling sites [22], assessment methods [23], reliance on dietary survey instruments with inherent recall bias, and the use of short- versus long-term dietary interventions to study intestinal microbiota and metabolite composition [12,14].

Animal models that utilize dextran sodium sulfate (DSS) to induce colitis have been informative for studying human UC because they reproduce most features of the disease, including inflammation, diarrhea, and abnormal microbial composition in feces [24]. The DSS model has been extensively used to investigate the role of gut microbiota in IBD development and the influence of environmental factors such as diet on colitis severity. DSS disrupts mucosal barrier integrity, allowing pro-inflammatory bacteria to penetrate underlying tissues. These pathobionts stimulate the innate lymphoid response, triggering an influx of inflammatory cells and actively contributing to disease pathogenesis, as DSS-induced inflammation is reduced in their absence [25]. The anatomical size and structure, physiology, immunology, and genome of pigs closely resemble those of humans, enhancing their potential as a translational animal model [26,27,28]. Like humans, pigs are omnivores [29], consume a similar amount of daily calories, use the colon—rather than the cecum—as the main fermentation site of fibrous dietary components, and have comparable digesta transit times and nutrient absorption [30]. DSS-induced colitis in swine resembles active IBD in humans [31], with crypt destruction and mucosal erosion [32], both influenced by intestinal microbial composition or diet [33,34,35]. Several studies examining risk factors for IBD have shown that consumption of FVs may lower the risk of developing UC; however, the mechanisms underlying this protective effect remain unclear [36]. We investigated the relationship between FV intake and colitis by supplementing a translational pig model with FVs at DGA-recommended levels. By integrating transcriptomic, histological, and metagenomic data, we employed a nutrigenomic approach to map the complex pathways connecting dietary habits to host health.

## 2. Materials and Methods

### 2.1. Animals and Diets

**Figure 1:**
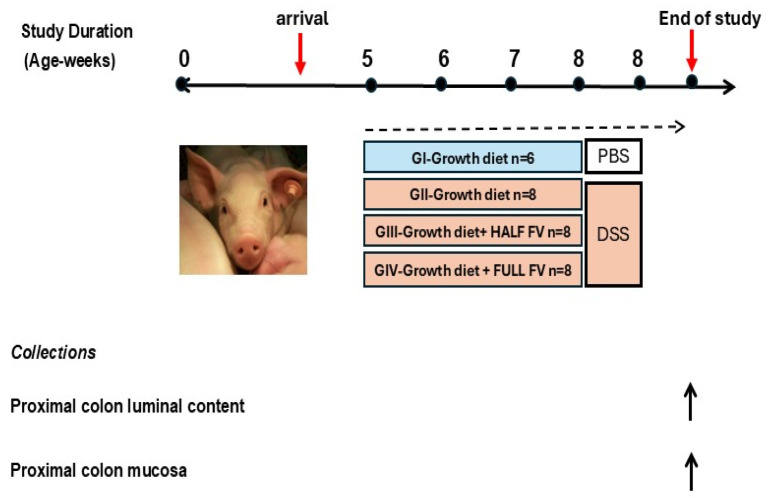
Experimental design. Thirty-three-week-old piglets were randomized by weight and allocated to four dietary groups: GI-Control (*n* = 6), GII-DSS-treated control (*n* = 8), GIII-half-FV-DSS (*n* = 8) and GIV-full-FV-DSS (*n* = 8). After one week of acclimatization, pigs started consumption of experimental diets for three weeks before receiving an oral challenge with 4% dextran sodium sulfate for 10 consecutive days to induce colitis (GII, GIII and GIV). Pigs were euthanized four days after the last DSS challenge. Samples from proximal colon content and mucosa were collected at the end of the study for further processing.

All animal experiments and husbandry procedures, including environmental enrichment, were conducted in accordance with guidelines approved by the Beltsville Area Animal Care and Use Committee (Protocol 19-016). Fresh or frozen fruits (green seedless grapes, strawberries, red apples, blackberries, and blueberries) and vegetables (celery, broccoli, spinach, green beans and kale) were purchased from a single local market chain. All items were processed, cut, and steam-cooked (except celery and fruits) according to the manufacturer’s instructions. The fruits and vegetables (FVs) were weighed separately before and after freeze-drying to calculate the weight required for an equal volume contribution of each (Table S1. swine experimental diet composition). These calculations were based on a daily intake of 2.5 cups of fruits (52 gr) and 3.5 cups of vegetables (22 gr), as recommended by the Dietary Guidelines for Americans (DGA) for healthy adults between the ages of 19 to 59 years consuming a 2800-kilocalorie diet (www.dietaryguidelines.gov). Additionally, two 2-ounce servings of chicken breast were sliced, microwaved according to the manufacturer’s instructions, and freeze-dried before being incorporated into all diets (14 gr). This contributed approximately 50% of the recommended daily animal protein. The macronutrient composition of dietary ingredients was determined by the Eurofins Nutritional Analysis Laboratory (Eurofins Scientific Inc, Des Moines, IA, USA) to formulate diets with similar calorie content (Table S1). Twenty-one piglets from pregnant Yorkshire x Landrace sows (Oakhill Genetics, Ewing, IL, USA) that farrowed at swine facilities in Beltsville, MD, were used for a pilot study to determine the appropriate dose of DSS for inducing clinical colitis within humane endpoints. For the main experiment, a power analysis was conducted using hypothetical gene-response scenarios based on variability observed in previous studies involving dietary supplementation with and without inflammation. These scenarios represented minimal biologically meaningful effects that the statistical model should detect if they occurred in the study. The power analysis indicated that six to eight pigs per treatment would produce an 85–95% probability of detecting the expected dietary effects as statistically significant. The main experiment involved thirty healthy female pigs (Landrace X Yorkshire), all approximately six weeks old, obtained directly from Oakhill Genetics. Prior to shipment, inclusion criteria were established to ensure uniformity in age and body weight among animals, thereby reducing variability in the study. To minimize potential confounder factors, pig weights (collected the day before delivery) were balanced across four experimental treatment groups (*n* = 6–8 pigs per group). Within each group, littermates were distributed across different dietary treatments to maximize genetic diversity and maintain a balanced weight and age range of up to six days. Upon arrival, pigs were housed individually in identical pens at the Beltsville Agricultural Research Center, Beltsville, MD. Each pen was equipped with solid partitions to prevent direct contact between animals while maintaining similar environmental conditions. All pigs had ad libitum access to drinking water and were provided isocaloric diets formulated to contain 16% protein, along with vitamins and minerals, in accordance with the National Research Council (NRC 2012) guidelines for optimal growth. Pigs were fed a regular age-matched growth diet ad-libitum for the first week after arrival for acclimatization before gradually switching to a daily pre-weighed feed amount (750 grs/day) of growth diet supplemented with chicken and fruits and vegetables (FVs) according to treatment group assignment (half or full FV-dose) or a calorie-matched, no-FV growth diet for the two control groups (Groups I–IV, Figure 1). All growth diets provided 17% energy (E) from fat, 62% E from carbohydrates, and 20% E from protein, with total dietary fiber ranging from 9.6% to 10.6%, depending on the diet. Dietary groups were designated as the untreated negative control diet (Group I, *n* = 6) and the DSS-treated positive control (Group II, *n* = 8). Group III (*n* = 8) and Group IV (*n* = 8) received the same grower diet supplemented with either half or the full daily fruit and vegetable (FVs) recommendations according to the DGA, respectively. All feeders were colored-labeled to display dietary group assignments. In addition, a regular age-matched growth diet was offered ad libitum to all treatment groups after consumption of the pre-weighed daily amount to meet or exceed growth nutrient requirements. Feed consumption was monitored by visually checking feeders daily and weighing their contents at least three times a week for dietary intake calculations and compliance. All diets contributed similar calories from macronutrients but with differences in carbohydrate and fiber sources. At the end of week 4, DSS (MW 36–50 kDa, MP Biomedical, Santa Ana, CA, USA) was dissolved in phosphate-buffered saline (PBS)(Quality Biologicals, Gaithersburg, MD, USA) to 4% weight/volume before orally administered to overnight-fasted pigs using a feeding cannula to induce moderate clinical disease, including diarrhea but without profuse bleeding or loss of appetite (within the humane endpoint under the animal protocol), based on pilot study observations and previous reports with growing pigs [33,37]. The first oral daily 4% DSS dose was dissolved in 200 mL of PBS (day 1), followed by 4% DSS dissolved in 100 mL for nine additional days (day 2–day 10), completing a 10-day DSS treatment for pigs in Groups II, III, and IV, while Group I received an equivalent volume of PBS. Daily fecal samples were collected after the DSS challenge. All thirty pigs completed the study with no exclusions. PC contents and tissue were collected at the end of the six-week dietary intervention (four days after the last DSS dose). All pigs were euthanized by IV injection of Euthasol (50 mg sodium pentobarbital/kg of body weight; Virbac Animal Health, Inc., Fort Worth, TX, USA) at the end of the study.

### 2.2. Clinical Signs and Disease Assessment

Pigs were monitored daily throughout the study to evaluate the effects of diet and DSS treatment on their growth and clinical signs of colitis, in accordance with humane endpoints (avoiding profuse bloody diarrhea, loss of appetite and moribund condition) as specified in the approved animal protocol. Feed intake was recorded daily and body weight was measured bi-weekly in a blinded manner using ear tags as identifiers. Body weight was assessed at baseline, at weeks 2 and 4 following FV intervention (prior to DSS treatment), and at weeks 5 and 6. Stool consistency was visually evaluated and scored using ear tags identifiers following scale: 0: formed stools; 1: slightly formed; 2: dense, not formed; 3: loose, not formed; and 4: watery stools/diarrhea. Fecal occult blood was assessed in properly labeled fecal samples collected daily from day 5 through day 13 after the DSS challenge using the guaiac Beckman Coulter Fecal Occult Blood Test (FOB) (Beckman Coulter, Brea, CA, USA). The intensity of color change was scored on a scale from 0 to 4, as previously described [38]. The individual disease activity index (DAI) was calculated by adding the stool consistency score and FOB score and was used to evaluate the grade of colitis [38].

### 2.3. PC Microscopic and Histopathological Analysis

Sections of PC, obtained from the same anatomical region, were collected for metagenome analysis and histopathology evaluation after fixation with 10% buffered formalin immediately after collection. Fixed sections were processed, embedded, sectioned, and stained with hematoxylin and eosin for histological evaluation by the Comparative Pathology Shared Resource Center at the University of Minnesota, St. Paul, MN, USA. All tissues were blindly evaluated by a certified veterinary pathologist. Measurements from PC sections and mucosal surface area were used to calculate the percentage of each section that consisted of mucosa, which informed possible differences in mucosal depth. Within the mucosal surface area, the percentage of erosion was also calculated using digitalized images of the PC where the full length of the lumen was visible. A subjective grading scale of 0–4 (0 = absent, 1 = minimal, 2 = mild, 3 = moderate, 4 = marked) was applied to the following features: inflammatory cell infiltrates (predominantly mixed mononuclear cells and some polymorphonuclear cells; “infiltrates”), crypt hyperplasia (“CH”), goblet cell hyperplasia (“GC”) and increased amount of mucus (“mucus”). These grades were used for comparison of mucosal changes among treatment groups.

### 2.4. PC Mucosa (PCM) Transcriptome Analysis

Whole PC sections (approximately 5 cm wide) were excised, opened with scalpels and forceps, and gently rinsed with cold sterile phosphate-buffered saline (PBS). The PCM layer was cleanly separated from the submucosal layer using a sterile scalpel and tweezers. PCM sections were collected in numbered and labeled cryotubes, immediately flash frozen in liquid nitrogen and stored at −80 °C until they were processed in one batch for RNA isolation. Total RNA from PCM was extracted using the TRI reagent according to the manufacturer’s instructions (Zymo Research, Irvine, CA, USA). Residual genomic DNA was removed using the TURBO DNA-free kit (Invitrogen, Carlsbad, CA, USA) with RNAseOUT (Invitrogen, Carlsbad, CA, USA). RNA quality and concentration were assessed by gel electrophoresis using the Experion RNA Standard Sensitivity Analysis Kit (Bio-Rad, Hercules, CA, USA). All samples had an RNA quality indicator (RQI) greater than 8.5. RNA sequencing libraries were prepared with Illumina TruSeq RNA Sample Prep v2 kits (Illumina, San Diego, CA, USA) according to the manufacturer’s protocol, as previously described [39]. The combined library pool was denatured and loaded on an Illumina Next Seq 500 sequencer (Illumina, San Diego, CA, USA), using a high-output flow cell to generate 150 base-pair single-end reads. Base-call conversion, de-multiplexing and adapter trimming of the pooled sequence data were performed using bcl2fastq2 conversion software (v2.20.0.422, Illumina, Inc.). Unaligned FASTQ files generated from sequencing were imported into CLC Genomics Workbench version 11.0 (QIAGEN, Bioinformatics, Redwood City, CA, USA), where they were trimmed to remove low-quality reads and adapter sequences. Reads were mapped to a custom non-redundant (NR) (NR_111819 v2) database containing 8959 transcript library (all sequences are found in the Porcine Translational Research Database (Harrys-Mac-Studio.local) [40], maintained by the Beltsville Human Nutrition Research Center, Diet, Genomics, and Immunology Laboratory, which includes curated sequences of genes involved in immunity/inflammation and nutrient metabolism, as previously described [41,42]. Mapped reads (including gene variants) for each sample were used to generate gene-level expression counts, which were used as input for differentially expressed gene (DEGs) analysis.

### 2.5. PC Content DNA Extraction and Library Preparation

PC contents were collected in 5 g numbered and labeled cryotubes at the end of the experiment, immediately frozen in liquid nitrogen, and stored at −80 °C until further processing. Samples were processed at Microbiome Insights (Vancouver, BC, Canada) for DNA extraction using the Qiagen MagAttract PowerSoil DNA KF kit (Qiagen, CA, USA), according to the manufacturer instructions. DNA quality was assessed via gel electrophoresis and quantified using a Qubit 3.0 fluorometer (Thermo Fisher, Waltham, MA, USA). Libraries were prepared using Illumina Nextera Library kits following the manufacturer’s protocol (Illumina, San Diego, CA, USA). Paired-end sequencing (150 bp × 2) was performed on a Next Seq 500 in medium-output mode. Shotgun metagenomic sequence reads were processed using the Sunbeam pipeline. Initial quality assessment was conducted with FastQC v0.11.5 (https://www.bioinformatics.babraham.ac.uk/projects/fastqc/) (accessed on 27 January 2021). Adapter sequences were removed using cutadapt v2.6 (https://cutadapt.readthedocs.org/ accessed 27 January 2021), and trimming was performed with Trimmomatic v0.36 using custom parameters (LEADING:3 TRAILING:3 SLIDINGWINDOW:4:15 MINLEN:36) [43]. Low-complexity sequences were detected with Komplexity v0.3.6 [44]. High-quality reads were mapped to the swine genome (Sscrofa11, GCF_000003025.6), and those with at least 50% similarity across 60% of the read length were removed from further analysis. The remaining reads were taxonomically classified using Kraken2 with the PlusPF database from 27 January 2021 [45]. For functional profiling, high-quality filtered reads were aligned against the SEED database via translated homology search and annotated to Subsystems or functional levels 1–3 using Super Focus [46]. Functional profiles were also aligned using the Uniref database and HUMAnN2, where the gene families were grouped into other ontologies [47]. Gene families annotated to metabolic enzymes were further analyzed to reconstruct and quantify complete metabolic pathways (i.e., MetaCyc) within the community.

### 2.6. Statistical Analysis

Clinical signs scores for tracking weight, fecal occult blood, diarrhea and proximal colon microscopic changes from all thirty pigs were compared using repeated measures- ANOVA models to accurately estimate variability among pigs and variability associated with repeated measurements within pigs across days. Because the observed among-pigs within-treatments variability was not homogeneous for all treatments and days, the ANOVA models were specified to accurately estimate the observed heterogeneous variances (i.e., greater than 2× difference in magnitude) associated with each treatment x day by employing the GROUP = option in the SAS PROC MIXED REPEATED statement (SAS for Windows, v9.4 TS1M8, SAS/STAT v15.3, Cary, NC, USA). As a result, these models appropriately partitioned the observed data variability to obtain correct statistical inferences. Proximal colon mucosa gene counts derived from thirty pigs in each dietary treatment were used to identify differential gene expression. Sequences with a quality score below Q30 or reads containing more than two ambiguous nucleotides were removed before sequence alignments were performed using CLC Genomics Workbench version 12 (Qiagen Bioinformatics, Redwood City, CA, USA). Gene expression was normalized using the “reads per Kilobase of exon-model per million mapped reads” (RPKM) method [48]. Differential gene expression analysis was carried out using the Bioconductor package DESeq2 v 3.14 [49]. Genes with an absolute fold-change ≥ 1.4 and a false discovery rate (FDR)-adjusted threshold of <0.05 relative to the control group were considered differentially expressed genes (DEGs). Permutational multivariate analysis of variance was used to estimate the effects of experimental treatments on proximal colon taxonomic and functional profiles and pairwise comparisons among treatment groups. A negative binomial model implemented in the DESeq2 v 3.14 R package was also used for differential abundance testing of taxonomic and subsystem-level 3 functional features. *p*-values were calculated and adjusted with the likelihood-ratio test.

## 3. Results

### 3.1. Clinical Signs

**Figure 2:**
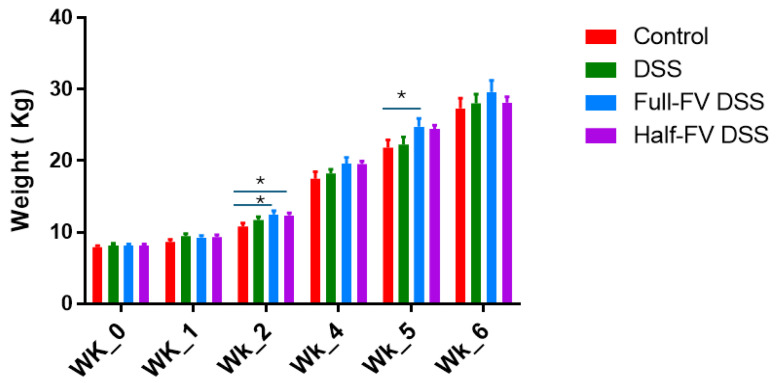
Weekly weight. Pig body weight was recorded at baseline, every two weeks during dietary intervention, and weekly following DSS challenge beginning at the end of week 4. Bars represent mean body ± standard error (SE) for pigs in negative control, DSS-positive control, half-FV-DSS, and full-FV-DSS dietary groups. Group means were compared using an ANOVA model, and significant differences identified through pairwise comparisons (*p* < 0.05) are indicated by an asterisk (*).

The pigs were clinically healthy, alert and showed no adverse treatment effects on appetite. All thirty pigs gained weight according to age-matched expected growth curve, with increased weight gain observed in both FV-supplemented groups two weeks after dietary intervention and at week 5 (one week after DSS challenge) for the full-FV-DSS group relative to the control group (*p* < 0.1) (Figure 2) (Table S2. clinical measures). This transient increase in weight was attributed to greater consumption of the ad libitum regular growth diet after the pre-weighted (750 grs) treatment diets were administered to maintain the intended daily FV dose (Figure S1. feed consumption).

**Figure 3:**
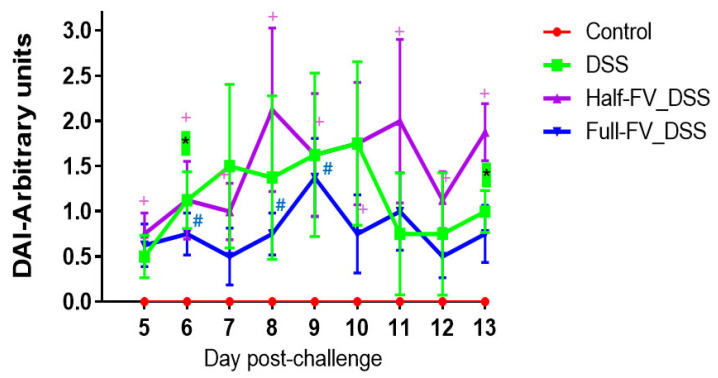
Disease activity index. Fecal occult blood (FOB) and stool consistency scores from samples collected between days 5 and 13 post-DSS challenge were used to assess differences in clinical disease among treatment groups. A two-way repeated-measures ANOVA (treatment X day), as described in Materials and Methods, was applied to model variation across days within individual pigs. Pairwise comparisons of treatment means at each day were conducted using the least significant difference (LSD) method by specifying SLICE = day in the LSMEANS statement. Significant differences are indicated by an asterik (*) for the DSS-positive control group, a plus sign (+) for the half-FV-DSS group, and a hash sign (#) for the full-FV-DSS group.

Following the DSS challenge, beginning in week 4, clinical signs and stool consistency were monitored daily. One pig in the DSS-positive treatment group (*n* = 8) exhibited persistent diarrhea for six consecutive days, commencing on day seven post-treatment. In the half-FV-DSS treatment group (*n* = 8), two pigs developed transient diarrhea, each for a single day, occurring on days eight and ten post-treatment, respectively. No alterations in stool consistency were observed in pigs assigned to the full-FV-DSS (*n* = 8) or negative control (*n* = 6) groups throughout the study period. Notably, digested blood was intermittently detected in formed feces from multiple pigs across all DSS-treated groups starting on day five post- challenge. Fecal occult blood (FOB) and stool consistency scores were utilized to calculate the disease activity index (DAI), which was compared among treatment groups from day 5 through day 13 post-challenge (Table S2). Relative to the non-DSS control group, DAI values were significantly elevated for two and nine days in the DSS and half-FV-DSS groups, respectively, and for three days in the full-FV-DSS group (*p* < 0.05) (Figure 3).

### 3.2. Morphometric Analysis of PC Mucosa Indicated Dietary Induced Differences in Tissue Response to DSS Treatment

**Figure 4:**
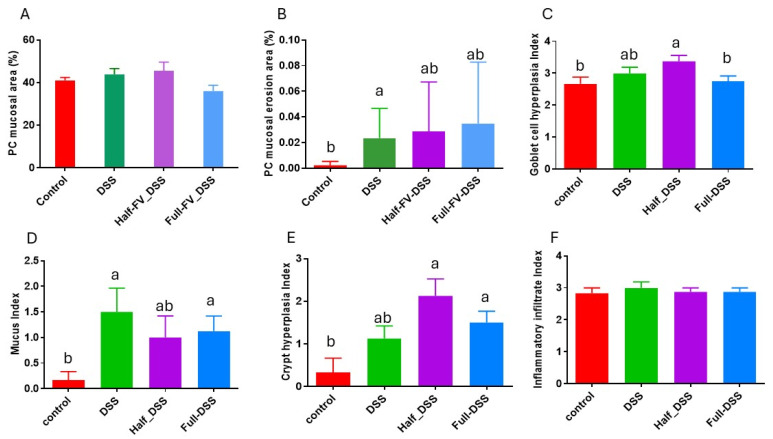
Morphometric analysis of proximal colon mucosa. Sections were evaluated for mucosal area (%) (**A**) and mucosal erosion (%) (**B**) as described in Materials and methods. Goblet cell hyperplasia (**C**), mucus presence (**D**), crypt hyperplasia (**E**), and inflammatory cell infiltrates (**F**) were scored on a 0–4 scale. Bar represents group means ± SE for negative control (red), DSS-positive control (green), half-FV-DSS (purple), and full-FV-DSS (blue). Significant differences vs. negative control are indicated by different superscript letters (*p* < 0.05).

Blinded evaluation of all thirty digitalized PC histological sections by a pathologist revealed no significant differences in mucosal area among treatment groups (Figure 4A) (Table S3. morphometric analysis of PC mucosa). However, the DSS-positive group (*n* = 8) exhibited a higher percentage of mucosal erosion (0.023 ± 0.008) compared to the negative control (*n* = 6) (0.002 ± 0.001) (*p* < 0.05) (Figure 4B). The mean goblet cell hyperplasia score was significantly increased in the half-FV-DSS group (*n* = 8) (3.37 ± 0.18) relative to the negative control (*n* = 6) (2.66 ± 0.21) and the full-FV-DSS group *(n* = 8) (2.75 ± 0.18) (*p* < 0.05) (Figure 4C). Mucus production was also elevated in the DSS-positive (1.50 ± 0.44) and full-FV-DSS (1.12 ± 0.29) groups compared to the negative control (0.16 ± 0.17) (*p* < 0.05) (Figure 4D). Multifocal areas of epithelial regeneration, crypts distended with mucus, and plump, tortuous crypt morphology were observed in two of eight pigs in the DSS group, four of eight in the half-FV-DSS group, and all pigs in the full-FV-DSS group, suggesting prior mucosal damage with varying degrees of regeneration among groups. A significant increase in PC crypt hyperplasia grade was detected in the half-FV-DSS (2.12 ± 0.40) and full-FV-DSS (1.5 ± 0.28) groups compared to the negative control (0.03 ± 0.33) (*p* < 0.05), whereas no significant difference was observed in the DSS-positive group (1.12 ± 0.28) after pairwise comparisons among all groups (Figure 4E). No significant differences were detected in mucosal cell infiltrates among treatment groups (Figure 4F) (Table S3).

### 3.3. Fruit and Vegetable Dietary Supplementation Modulated PC Transcriptome Response to DSS-Induced Colitis

**Figure 5:**
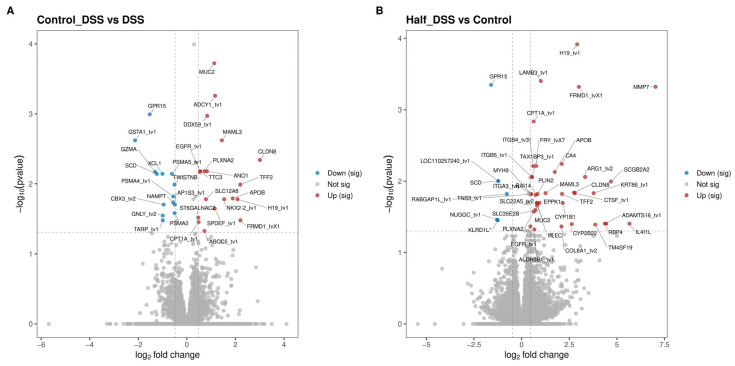
Differentially expressed genes in proximal colon mucosa (PCM). The PCM transcriptome of pigs from DSS-treated control and half-FV-DSS groups was compared to that of the negative control diet. Volcano plots depict the fold difference of 41 differentially expressed genes (DEGs) in the DSS-treated control group (**A**) and 52 DEGs in the half-FV-DSS group (**B**), relative to the negative control group. No DEGs were detected in the full-FV-DSS group. An absolute fold-change threshold of 1.4 with an adjusted FDR ≤ 0.05 was applied to identify significant gene expression differences (red dots represent upregulated genes, blue dots for downregulated genes). Only the most significant DEGs are shown in the volcano plots for the DSS-treated control and half-FV-DSS groups.

RNA derived from proximal colon mucosa was processed for all thirty pigs in control and FV-supplemented diet groups as independent replicates for read mapping. Principal component analysis (PCA) using the NR gene count dataset did not show separation between dietary groups (Supplementary Figure S2). Differential gene expression analysis was performed using the DESeq2 v.3.14 Bioconductor package. Volcano plots of the PCM transcriptome indicated that DSS-induced changes in gene expression, compared to the negative control group, were influenced by dietary treatment. These changes included 34 differentially expressed genes (DEGs), (19 upregulated, 15 downregulated, FC ≥ 1.4, FDR < 0.05) in the PCM of pigs exposed to DSS (*n* = 8) (Figure 5A). In pigs fed the half-FV diet (*n* = 8) after DSS exposure, 45 DEG were identified (40 upregulated, 5 downregulated, FC ≥1.4, FDR < 0.05) (Figure 5B). No DEGs were detected in the full-FV-DSS group *(n* = 8) relative to the negative control group (*n* = 6) (Table 1). When FV-supplemented groups (half-FV-DSS and full-FV-DSS) were compared with the DSS-treated positive control group, only two DEGs (*CXCL10*, *TTC3*) were found in the full-FV-DSS group, and none were detected in the half-FV-DSS group (Figure S2).

### 3.4. Fruit and Vegetable-Supplemented Diet Affected PC Metagenome

**Figure 6:**
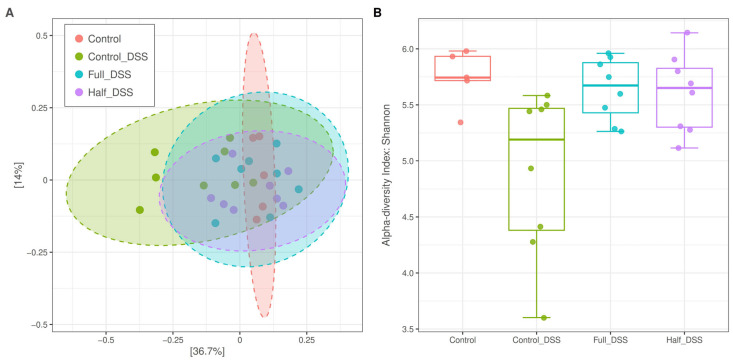
Proximal colon content microbiome diversity. Principal coordinate analysis with each unique symbol described in the legend representing the proximal colon microbiome from pigs in the negative control diet (red), DSS-treated control (green), half-FV-DSS (blue), and full-FV-DSS (purple) groups, was used to compare all microbial communities based on their composition (beta diversity) using the Bray–Curtis index and pairwise PERMANOVA to test for statistical significance. (**A**). Microbial alpha diversity (Shannon index) distribution values are summarized with a line representing the median and diamond the mean at the species level. Significant difference after pairwise comparisons against the negative control denoted by different superscript letters (FDR < 0.05) (**B**).

Sequence quality was high, and host contamination was generally low, with a median contamination of 1.5%. One sample from the negative control group was removed due to excessive host contamination. Feature abundance tables were derived from 29 libraries, with an average size of 658,970 ± 258,416 reads (mean ± SD). Principal coordinate analysis (PCoA) was performed to compare microbial communities based on beta diversity using the Bray–Curtis Index, and pairwise PERMANOVA was applied to test statistical significance with multiple testing adjustment. Pairwise comparisons revealed significant differences in diversity between DSS-negative (*n* = 5) and DSS-positive (*n* = 8) control groups (FDR < 0.05) and between DSS-negative and half-FV-DSS (*n* = 8) groups (FDR < 0.1), but not the full-FV-DSS (*n* = 8) group, for taxonomic profiles. Microbial communities also differed between FV-DSS-treated groups and the DSS-positive control group (FDR < 0.05; PERMANOVA F = 2.65; R-squared: 0.24; *p* = 0.003) (Figure 6A). Alpha diversity at the species levels, measured by the Shannon diversity index, was reduced in the DSS-positive control group (Kruskal–Wallis chi-squared= 8.88, df = 3, *p* = 0.04) (Figure 6B). Pairwise comparisons indicated significant differences between the DSS-positive control group and the DSS-negative control group, as well as both FV-supplemented DSS-treated groups.

Bacteria dominated the metagenome communities, followed by Archaea, Eukaryotes, and viruses, accounting for 96.8%, 2.4%, 0.8%, and 0.04% respectively. Taxonomic abundances within each community were visualized using stacked bar plots. Bacterial communities were primarily composed of Firmicutes, Bacteroidetes and Proteobacteria (Figure S3a, Bacterial composition); Archaea were dominated by Methanobacteria (Figure S3b, Archaeal composition); and Eukaryotes by Ascomycota (Figure S3c, Eukaryote composition). Differential abundance testing was performed at multiple taxonomic levels. Compared to the negative control (non-DSS treated group), there was an overall increase in Saccharomyces abundance, reflected by more than a two-fold increase at the phylum Ascomycota level, a three- to six-fold increase at the class (Saccharomycetes) and order Saccharomycetales levels, and more than a four-fold increase at the family *Saccharomycetaceae* level across all DSS treatment groups. Additionally, a two-fold increase was observed in *Debaryomycetaceae*, *Pichiaceae*, *Trichomonascaceae* in both FV-supplemented groups, and in *Phaffomycetaceae* exclusively in the full-FV DSS group (Table 2). A four-fold increase in the phylum Euryarchaeota and a five-fold increase in the class Methanobacteria were detected in the DSS-positive control and half-FV-DSS groups only. In contrast, a 22-fold and 5-fold increase in the abundance of the families *Chlamydiaceae* and *Desulfovibrionaceae,* respectively, was observed exclusively in the half-FV-DSS group (FDR < 0.05) (Table 2). Relative to the DSS-positive control group, the half-FV DSS group also exhibited a five-fold increase in *Elusimicrobiaceae* and a four-fold increase in *Desulfovibrionaceae,* whereas the abundance of *Desulfovibrionaceae* was reduced in full-FV-DSS group compared to the half-FV-DSS group (Table 2).

**Figure 7:**
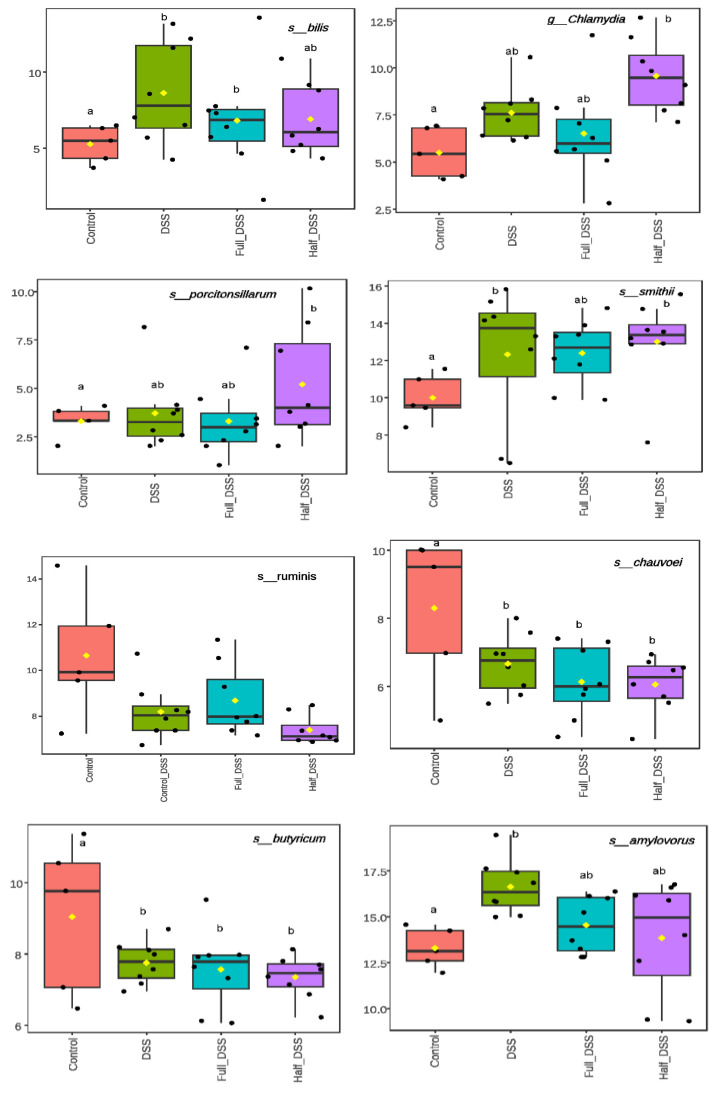

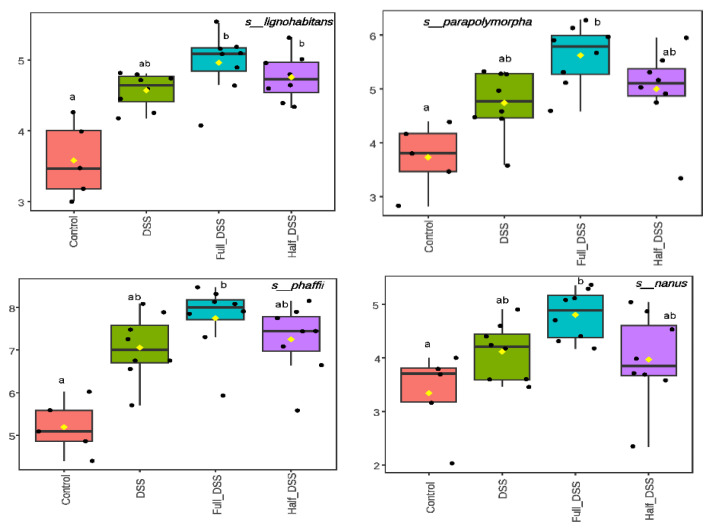
Differential species abundance in proximal colon contents. Representative bacterial species abundance counts after logarithmic transformation are shown. Boxplots display the distribution of Log_2_ values, the mean with yellow diamond, with a line indicating the median for each treatment group: negative control (pink), DSS-positive control (green), half-FV-DSS (purple), and full-FV-DSS (blue) treatment groups. Different letter superscripts indicate significant differences in abundance among groups (FDR < 0.05). The full set of differential species is summarized in Supplementary Table S4).

Pairwise comparisons for differential abundance testing at the species level were calculated relative to negative and positive DSS control groups, and *p*-values were adjusted using the LRT test. Differential abundance was detected for 101 species (FDR < 0.05; Supplementary Table S4). DSS treatment induced changes in the abundance of several species within Proteobacteria. Most notably, there was a 28- to 34-fold increase in pathogenic bacteria affecting the gastrointestinal tract, including *Helicobacter bilis*, in the DSS-positive control and full-FV-DSS groups. Additionally, a 28-fold increase in pathogenic *Chlamydia suis* and a 17-fold increase in the commensal *Actinobacillus porcitonsillarum* were observed exclusively in the half-FV-DSS group (Figure 7). Within Archaea, there was an eleven-fold increase in *Methanobrevibacter smitthii* in the DSS-positive control and half-FV-DSS groups, accompanied by a reduction in the abundance of other *Methanobrevibacter* species in the full-FV-DSS group relative to the control group (Table S4). Among Firmicutes, several Clostridium genera showed reduced abundance across all DSS-treated groups. Specifically, *Ligilactobacillus ruminis* exhibited a 17-fold and 43-fold reduction in the DSS-positive control and half-FV-DSS groups, respectively. Conversely, an increase in several *Lactobacillus* species was observed exclusively in the DSS- positive control group, with no changes detected for any of these genera in the full-FV DSS group (Figure 7). Relative to the negative control group, differential taxonomic analysis revealed more than a four-fold increase in 19 species within the phylum Ascomycota across all DSS-treated groups, along with three additional species present in both FV-supplemented DSS groups and six species unique to the full-FV -DSS group (Table S4). When species abundances in the FV-treated groups were compared to the DSS-positive control group, a four-and three-fold reduction in *Megasphaera elsdenii* and *Lactobacillus paragasseri* (phylum Firmicutes), respectively, was observed, along with a five-fold increase in *Elusimicrobium minutum* (phylum Elusimicrobia) in the half-FV-DSS group. In contrast, *Methanobrevibacter* sp. *ABM4* abundance decreased by 50-fold in the full-FV-DSS group (Table S4). No significant differences in species-level abundance were observed between the two FV-treated groups.

For functional profiling, reads were aligned to the SEED database using a translated homology search and annotated to Subsystem functional levels 1–3, focusing on microbial functions present in the input. The SEED database organizes subsystems into three hierarchical levels, with level 1 being the most general and level 3 the most specific, including functional roles. Differential abundance testing of level 3 functional features identified 47 unique functions when all DSS-treated groups were compared to the negative control or when FV-supplemented groups were compared to the positive DSS control group (Table S5, differential abundance of functional level features). Thirty-seven pathways were unique to the DSS control group compared to the non-DSS control group. These included changes in bacterial functions such as increased utilization of D-galactonate and mannitol as carbohydrate sources, reduced amino acid synthesis (glycine, serine, proline, threonine and histidine), increased amino acid racemase activity, enhanced formation of bacterial cell wall components through lipoteichoic acid biosynthesis and sialic acid metabolism, promotion of archaeal and eukaryotic cell components, and the presence of bacterial virulence factors, including antibiotic resistance genes, bacteriocins, and internalin-like proteins. Six unique functional pathways were identified in the half-FV-DSS group, including increased lysine degradation, beta-lactamase activity, and proteolysis pathways, while four pathways were unique to the full-FV-DSS group, including reduced citrate metabolism and increased sphingolipid biosynthesis compared to the non-DSS control group. Additionally, both FV-supplemented groups exhibited significant changes in carbohydrate metabolism pathways, such as reduced utilization of mannitol and lactate as fermentative products, alterations in amino acid and derivative pathways with decreased lysine fermentation and amino acid racemase activity, and changes in membrane transport pathways with reduced ABC transporter alkyl-phosphonate activity relative to the DSS-positive control group (Table S5).

**Figure 8:**
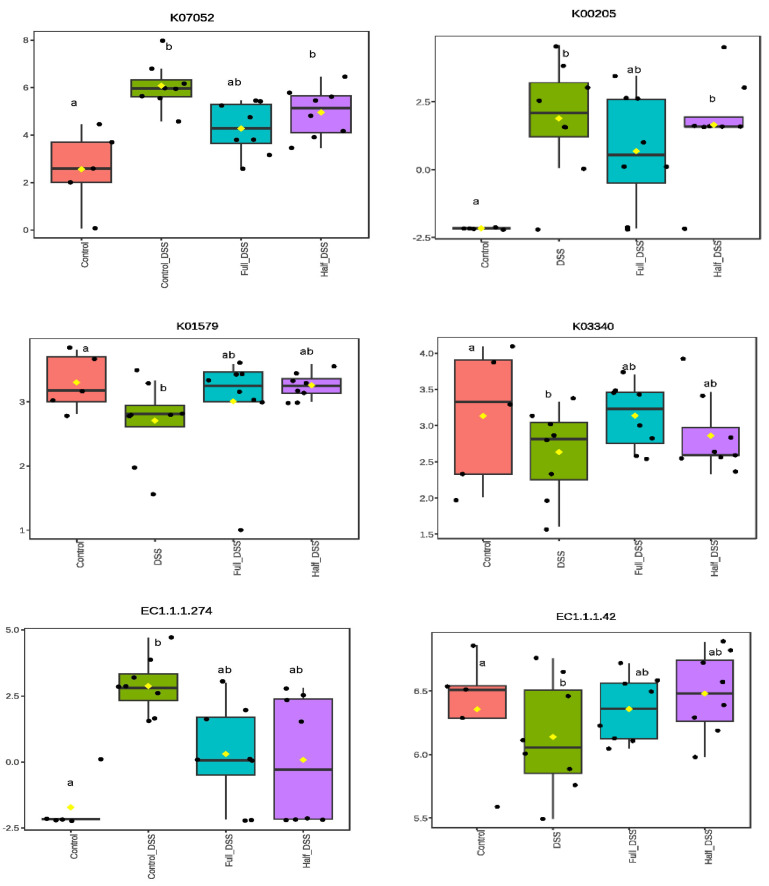

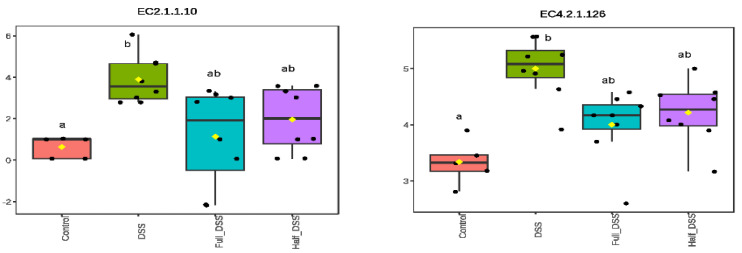
Differential functional pathway abundance in proximal colon contents. Representative functional KEGG pathways (KO) and enzyme (EC) abundance counts after logarithmic transformation are shown. Boxplots illustrate the distribution of Log_2_ values, with yellow diamond indicating the mean, a line indicating the median for each treatment group: negative control (pink), DSS-positive control (green), half-FV-DSS (purple), and full-FV-DSS (blue). Different superscript letters indicate significant differences in KEGG pathway or enzyme abundance among groups (FDR < 0.05). A complete list of differential pathways and enzymes is provided in Supplementary Table S6. (KO7052: peptidases and inhibitors, KO0205: methane metabolism, KO3340: lysine biosynthesis, K01579: beta-alanine metabolism. EC1.1.1.274: oxidoreductase, EC1.1.1.42: oxidoreductase, EC2.1.1.10: methyl-transferase, EC4.2.1.126: hydro-lyase.).

Functional pathways were profiled using KEGG orthologies (https://www.genome.jp/kegg/kegg2.html) and Enzyme Commission (EC) categories (https://iubmb.qmul.ac.uk/enzyme/) both accessed on 24 July 2025 to infer higher-level functions from metagenomic data (Table S6, differential abundance of KEGG and EC ontologies; Figure 8). Relative to the non-DSS control group, DSS treatment altered 103 KEGG pathways (FDR < 0.05). Among these, 31 pathways were upregulated (fold-change > 2)—notably methane metabolism (K00205, K00578), archaeal replication/repair (K02319, K07463, K10725), and carbohydrate utilization, including the citrate cycle (K01903) and starch/sucrose metabolism (K00690). Conversely, 52 pathways were downregulated, with decreases in oxidative phosphorylation (K00330), genetic information processing (replication/repair; K03773, K00561, K07481, K04762, K10947), membrane transport (K11720), and amino acid metabolism (K03340, K01579) (Table S6).

FV supplementation mitigated DSS-induced functional shifts in a dose-dependent manner. In the half-FV-DSS group, 15 KEGG pathways were affected, including upregulation of ABC transporters (K02009), methane metabolism (K00205), additional transporters (K07301), and glycerophospholipid metabolism (K00096), with downregulation in cofactor/vitamin metabolism (K16651) and transport-related cellular processes (K11733, K03756). In the full-FV-DSS group, no KEGG differences were detected versus the non-DSS control group, indicating normal function. However, relative to the DSS group, both FV-supplemented groups display significant shifts. The half-FV-DSS group showed reductions in carbohydrate metabolism (K01026, K02750, K18120, K01034) and transporters (K03756, K03313, K03535, K05786, K02042, K03293), whereas the full-FV-DSS group exhibited increases in pathways associated with carbohydrate metabolism (K00702, K01711, K01006, K01804, K00240, K00175, K00656), amino acid metabolism (K03340, K01579, K0290, K0812), and cofactors and vitamins (K01661, K03149, K06215, K09457) (Table S6).

EC-based enzyme abundance mirrored these trends. The DSS group showed 47 upregulated enzymes, predominantly oxidoreductases, peptidases (hydrolases acting on peptide bonds), and glycosylases. In the half-FV-DSS group, only one oxidoreductase was upregulated, and the full-FV-DSS group showed no changes versus the non-DSS control group. Relative to the DSS group, FV supplementation produced limited shifts—a reduction in several transferases and one lyase in the half-FV-DSS group, and numerous upregulated enzymes associated with the biosynthesis of secondary metabolites and amino acid metabolism in the full-FV-DSS group, consistent with dose-dependent functional recovery (Table S6).

## 4. Discussion

Differences in clinical signs, PC metagenome, PCM transcriptome and morphometric analysis demonstrated that a three-week daily dietary supplementation with 2.5 cups of fruits and 3.5 cups of vegetables, as recommended by the DGA, provided protection against experimental colitis. Pigs in the full-FV DSS group did not exhibit diarrhea, maintained gut microbiome diversity and metagenome function, and showed an unaltered transcriptome in PCM, although they exhibited increased PC crypt hyperplasia compared to control pigs, regardless of DSS-induced taxonomic differences in the microbiome. Pigs in the half-FV DSS group exhibited diarrhea with persistent FOB but maintained microbiome diversity and metabolic function. Additionally, this group showed goblet cell and crypt hyperplasia, along with a PCM transcriptome response associated with tissue remodeling and an increased host immune response. In contrast, pigs in the DSS-positive control group exhibited a mild but significant increase in mucosal erosion, reduced expression of genes required for detoxification and immune response in PCM, and decreased microbiome diversity. They also showed multiple changes in metabolic pathways affecting carbohydrate metabolism, amino acid biosynthesis, and cellular transport, along with an increase in virulence factors. Therefore, our data suggests that a full-FV diet provides strong protection against DSS-induced colitis, while a half-FV diet offers partial protection.

Healthy eating, including the consumption of fruits and vegetables (FVs), has been shown to modify microbiome composition and function in healthy individuals [50] and in colorectal cancer survivors, with a protective role against clinical recurrence [51,52]. Previously, controlled feeding of a diet containing half the daily recommended DGA level for FVs to pigs for two weeks induced differentially expressed genes (DEGs) in whole blood cells, with increased B-cell function and cellular movement. These changes correlated with alterations in the gut microbiome, including increased abundance of fermentative bacteria associated with improved intestinal health [39]. In this follow-up colitis study, the inflammatory response was modulated by dietary intervention, with reduced or absent clinical signs in FV-supplemented groups. Further histopathological evaluation indicated greater reactivity in both FV-treated groups. Increased intestinal mucosa cell turnover could explain the observed crypt depth, which has been linked to enhanced bacterial fermentation, as previously reported with diets containing increased fiber levels [53,54,55]. In our study, FV-supplemented diets provided an additional daily 11 and 5.6 g of predominantly insoluble fiber per every 750 g of diet consumed in the full-FV and half-FV diets, respectively, along with natural dietary polyphenols, including common flavonoids. In a corollary metabolomic study comparing in vivo metabolic profiles from fecal samples derived from the two FV-supplemented diets, common flavonoid-derived metabolites, epicatechin and protocatechuic acid, were identified as highly discriminating in fecal samples as early as one week after dietary intervention, in a dose-dependent manner, using a non-targeted metabolomic approach [56].These findings suggest that these and other metabolites present in the proximal colon may have prevented or alleviated PCM inflammation through multiple mechanisms, including inhibition of inflammation and oxidative stress, modulation of intestinal microbiota and immune response, and suppression of key inflammatory pathways such as NF-κB, as recently reviewed in in vivo and limited pre-clinical studies [57,58]. Gut microbes interact with metabolites, jointly regulating inflammatory responses, oxidative stress, and energy metabolism. Recent research indicates that specific metabolites and pathways are associated with ulcerative colitis disease progression [59,60]. Therefore, when FV-enriched polyphenols are combined with fiber, these compounds may synergistically improve gut health through modulation of gut microbiota [61] and bioactive signaling metabolites [62]. A longitudinal study with multiple time points, rather than a cross-sectional design, could better capture the extent of DSS-induced colitis damage in PCM crypt structure, as previous research suggests DSS-induced colitis peaks on day 5 and resolves by day 7 in five-week-old pigs [38]. Nevertheless, our histopathological findings at four days post-DSS exposure suggested a FV-induced regenerative effect that also influenced PCM transcriptome and metagenome responses.

**Figure 9:**
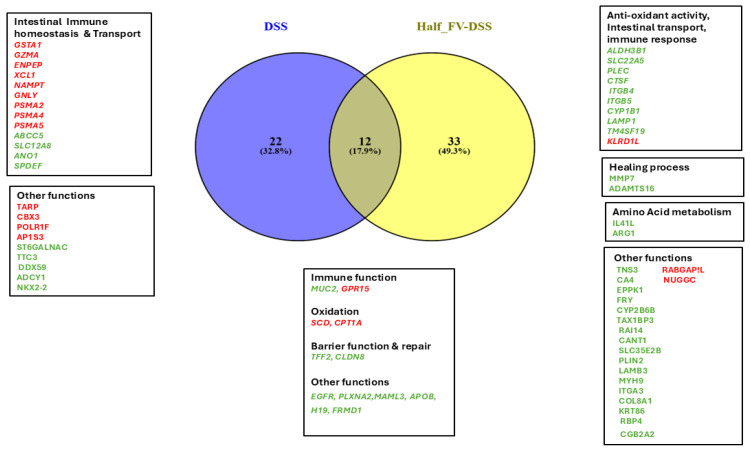
Venn analysis of differentially expressed genes in proximal colon mucosa of DSS-treated pigs. Differentially expressed genes with >1.4-fold-change (FC) at FDR < 0.05 in DSS-treated control and half-FV-DSS groups were analyzed using the online tool Venny 2.1 (https://bioinfogp.cnb.csic.es/tools/venny) accessed on 14 January 2026. Downregulated and upregulated genes relative to control groups are displayed in red and green fonts, respectively. Common genes (2 downregulated, 10 upregulated) and unique upregulated and downregulated genes in the DSS-treated control group (13 downregulated, 9 upregulated) and half-FV-DSS group (3 downregulated, 30 upregulated) were grouped according to their function. No DEGs were detected in the full-FV-DSS group at FDR < 0.05.

All significant differences in gene expression observed in the DSS and half-FV DSS-treated groups, with no DEGs detected in the PCM of the full-FV-DSS group relative to the negative DSS control, are summarized Table 1. The top common DEGs expressed in PCM that were upregulated between two- and eight-fold compared to the negative control group (FDR < 0.05, Figure 9) included mucin 2 (*MUC2*), a biomarker for diagnosing colonic inflammation in pigs [53]; claudin 8 (*CLDN8*), a marker for cation barrier function that seals the paracellular barrier [63,64]; and trefoil factor 2 (TFF2), a regenerating factor shown to promote intestinal epithelial cell repair in DSS-induced colitis in mice [65,66]. Both DSS-treated groups also exhibited more than a two-fold downregulation of G-protein-coupled receptor-15 (*GPR15*), a lymphocyte receptor that specifically mediates trafficking of regulatory and effector/memory T cells [67,68] and stearoyl-CoA desaturase (*SCD*), an enzyme involved in lipogenesis and mitochondrial fatty acid oxidation [69]. Interestingly, the DSS-positive control and half-FV-DSS groups showed unique DEGs that differentiated their responses to DSS-induced colitis from each other (Figure 9).

Genes involved in maintaining intestinal immune homeostasis through detoxification, such as glutathione S-transferase alpha 1, (*GSTA1*) [70,71,72]; immune response genes including granzyme A (*GZMA*) and glutamyl aminopeptidase (*ENPEP*) [73,74]; intestinal dendritic cell activation (small inducible cytokine subfamily C, member 1, *XCL1*) [75]; redox sensitive activation (nicotinamide phosphoribosyl-transferase, *NAMPT*) [76]; antimicrobial activity (granulysin, *GNLY*) [77]; and proteasome 20S components (*PSMA2, PSMA4*, *PSMA5*), which mediate non-lysosomal protein degradation linked to antigenic peptide production and apoptosis in colonocytes [78], were downregulated in the DSS-positive control group. In contrast, dietary supplementation with half-FV modulated the expression of genes associated with antioxidant activity, intestinal transport and anti-tumor effects, including aldehyde dehydrogenase 3B1 (*ALDH3B1*) [79]; carnitine/organic cation transporter 2 (*OCTN2*/*SLC22A5*), a high-affinity transporter for absorbing L-carnitine that supports butyric acid oxidation and colonic epithelium integrity [80,81]; plectin (*PLEC*), linked to barrier function and negatively correlated with colitis severity [82]; and cathepsin F (*CTSF*), which exhibits anti-tumor effects through regulation of antigen presentation [83], among others (Table 1). Unique DEGs associated with tissue healing were upregulated only in pigs receiving the half-FV diet, including matrix metalloproteinase 7 (*MMP7*)*,* involved in cell proliferation, differentiation, angiogenesis, wound repair, and immune modulation through macrophage and neutrophil recruitment [84], and ADAM metallopeptidase with thrombospondin Type 1 motif 16 (*ADAMTS16*), strongly expressed in goblet cells and colonocytes and reported to inhibit tumor cell proliferation [85]. Modulation of amino acid metabolism was evident exclusively in the half-FV group, with upregulation of interleukin 4 induced 1 (*IL4I1L*), a tryptophan-metabolizing enzyme that depletes tryptophan and other aromatic amino acids (phenylalanine, tyrosine) to generate immunomodulatory metabolites [86,87,88,89], and arginase 1 (*ARG1*), which converts L-arginine to ornithine and urea while reducing NO production [90,91] and is associated with protection against DSS-induced colitis [92,93]. Overall, genes related to intestinal homeostasis, transport and detoxification were compromised in the DSS group but positively modulated in the half-FV group, enhancing antioxidant activity, intestinal transport, mucosal healing, and amino acid metabolism. The lack of detectable DEGs in the full-FV diet group suggests that the full-FV dietary intervention did not induce major transcriptional changes under the tested conditions or, alternatively, exhibited subtle changes below the detection threshold. Nevertheless, this stability may indicate homeostasis or resilience of the host. A high intake of fruits and vegetables (FVs) is strongly associated with reduced incidence of UC, as reported in a recent umbrella review analyzing dietary factors linked to the incidence or progression of human IBD [15,36,94,95]. Similarly, UC patients who regularly consume FVs are less likely to experience active disease phases [52].

Commensal bacteria prevent pathogen colonization by competing for space and nutrients. When the mucosal barrier is compromised, they can become pathobionts, crossing the epithelium and triggering inflammation [1]. Studies on IBD consistently report dysbiosis, characterized by reduced microbial diversity, expansion of facultative anaerobes, and depletion of *Clostridium* species—key regulators of intestinal homeostasis—alongside increased *Enterobacteriaceae* [7,96,97,98]. In our DSS-induced porcine colitis model, microbial diversity was reduced in the DSS-positive control group, whereas both FV-supplemented groups showed no significant change compared to the negative control group. The lack of diversity shifts suggests that FV supplementation helped maintain gut microbiome stability. Metagenomic changes partially mirrored human IBD, with increased Proteobacteria, reduced Firmicutes (notably Clostridiales), and higher abundance of *Lactobacilli* and *Methanobrevibacter smiithii* (Table S4). The DSS and full-FV-DSS groups showed elevated *Helicobacter bilis*, detected in human IBD and DSS mouse models [99,100], while *Chlamydia suis*, associated with enteritis [101], and *Actinobacillus porcitonsillarum,* a commensal species [102], were enriched only in the half-FV group. Pathogenic disruption of the gut microbiota has been linked to depletion of microbe-derived fermentation products (e.g., butyrate from *Clostridiales*), promoting metabolic reorientation of differentiated colonocytes toward a skewed metabolism characterized by increased lactase release, low oxygen consumption, and elevated inflammatory factors [53].This shift can lead to increased lactate production [103,104], favoring *Lactobacillus* strains that thrive in this environment and may help reduce DSS-induced injury [38]. In our DSS-induced colitis model, we observed a generalized decrease in *Clostridium* species, including [38] the butyrogenic *C. butyricum* [104,105], known to ameliorate colitis in mice, and *Ligilactobacillus ruminis* [106,107], in both DSS and half-FV-DSS groups. Our findings align with previous pig studies where colonic lactate accumulation after DSS challenge was associated with reduced injury and increased abundance of *Bifidobacterium* species [38]. This suggests that the increase in *Lactobacillus* species observed in the DSS group may have occurred in response to lactate accumulation during the resolution stage of colitis once DSS treatment was discontinued.

Ascomycota and Basidiomycota are the most prevalent fungal phyla in the gut of healthy individuals and other mammals [108,109,110,111]. In the human gut, the fungal community is primarily composed of genera such as *Aspergillus*, *Candida*, *Debaryomyces*, *Malassezia, Penicillium*, *Pichia* and *Saccharomyces* [112]. Similar to bacterial communities, the gut mycobiome can be significantly influenced by diet—one of the most important factors shaping its composition [113,114,115,116,117]. Evidence indicates a direct relationship between intestinal fungal composition and dietary patterns, suggesting that targeted dietary interventions may help prevent fungal-related gastrointestinal diseases [118]. Mycobiome dysbiosis has been linked to IBD pathogenesis, with decreased levels of *Saccharomyces cerevisiae, Debaromyces*, *Aspergillus* and *Cladosporium* in fecal samples of IBD patients, alongside increased abundance of *Candida albicans* compared to healthy controls, suggesting competition interactions among fungal species in the gut [119,120,121,122]. In contrast, Saccharomyces exhibits a protective, anti-inflammatory role in IBD [119] and murine colitis models [123] by promoting probiotic growth and enhancing microbial metabolites [124], which have been shown to ameliorate inflammation in DSS-induced colitis [100]. In our DSS-induced porcine colitis model, we observed increased abundance of genera within *Saccharomycetaceae* in response to DSS and families *Debaryomycetaceae*, *Pichiaceae*, *Trichomonascaceae* and *Phaffomycetacea* in PC contents following DSS challenge with FV-supplemented diets, suggesting their involvement in colonic inflammatory responses. These findings align with reports of reduced bacterial diversity and increased fungal burden, with decreased fungal diversity in ulcerative colitis [98,119,125]. Notably, FV-supplemented groups exhibited higher abundance of additional *Saccharomycetaceae* species, including *Sugiyamaella lignohabitans* (a lignocellulose degrader producing antimicrobial molecules) [126,127], *Kluyveromyces lactis* (a lactose fermenter) [128], and *Torulaspora delbrueckii* (a producer of lactic and succinic acids) [129], which were absent in the DSS-only group. Furthermore, non-conventional methylotrophic yeasts from *Pichiaceae* (*Ogatacea parapolymorpha*, *Brettanomyces nanus*), *Phaffomycetacea* (*Komagataella phaffi*), *Debaryomycetaceae* (*Debaryomyces hansenii*), and *Aspergillaceae* (*Aspergfillus oryzae*), previously associated with probiotic properties and bioactive metabolite production [130,131], were enriched exclusively in the full-FV-DSS group. These results indicate a potential modulatory effect of full-FV-supplementation on mycobiome composition and function, warranting further investigation. Collectively, our data indicate that FV consumption modulates colonic mycobiome composition, potentially contributing to intestinal homeostasis. Previous studies have shown that fruits and their bioactive polyphenols promote gut microbe-derived metabolites that counteract dysbiosis by enhancing short-chain fatty acid and vitamin production [50,132,133,134] and reducing reactive oxygen species and inflammation [135,136]. FV intake also introduces diverse, functionality active microorganisms to the host [137]. However, the role of fungi- derived metabolites in fermentation and their interactions with bacteria remains unclear. Addressing this knowledge gap is critical to determine whether dietary interventions should specifically target fungi to influence inflammatory processes such as IBD.

Metagenomic sequencing of fecal samples from IBD patients revealed changes in metabolic pathways, including increased oxidative stress and nutrient transport, along with a reduction in pathways related to carbohydrate metabolism and amino acid synthesis [7,97]. Similarly, among the 47 unique altered pathways identified in our colitis model, the amino acid biosynthesis pathways for serine, histidine, threonine, homoserine, and proline were reduced in the DSS-treated group compared to the non-DSS treated control group, while amino acid racemase activity, mannitol utilization, and virulence factors were increased. In contrast, FV-supplemented diets induced an increase in arginine biosynthesis and a reduction in lysine fermentation, as well as decreased fermentation of lactate and mannitol utilization in both FV-supplemented groups compared to the DSS-treated group. These findings suggest that FV supplementation reverses DSS-induced changes in amino acid and carbohydrate metabolism by modulating microbiome composition, as mannitol production in lactic acid bacteria strongly depends on carbohydrate fermentation pathways [138,139]. Therefore, our data indicate that FV consumption also plays a role in shaping PC metagenome function.

The strengths of our study include the use of high-throughput transcriptome and shotgun metagenome sequencing to assess the untargeted effects of a controlled dietary intervention consisting of defined amounts of fruits and vegetables combined with animal protein, providing a calorie-dense diet comparable to that of humans and thereby facilitating translational results. In our colitis model, we identified bacterial dysbiosis characterized by reduced diversity and changes in the PCM transcriptome, both of which were modulated by FV dietary intervention. The lack of DEGs in PCM of the full-FV intervention group limited our ability to construct microbial–transcriptome networks when exploring the mechanisms of dietary interventions. An important limitation of our study was the use of a single time point for data collection. In our experimental design, pigs were exposed to DSS for 10 days, and samples were collected on day 14 to compare the host response during inflammation and healing while consuming FV-supplemented diets. Therefore, our findings reflect diet–metagenome–host transcriptome responses after epithelial and crypt regeneration had begun, rather than the acute response to mucosal injury. The absence of differentially expressed genes (DEGs) in the full-FV-DSS group suggests that this group experienced earlier recovery of colonic mucosa injuries or, alternatively, that full-FV treatment mitigated DSS-induced effects by modulating microbiome and metabolome composition.

## 5. Conclusions

Differential metagenome and transcriptome responses in matching PC contents and mucosa demonstrated that a three-week daily FV dietary supplementation—consisting of 2.5 cups of fruits and 3.5 cups of vegetables per DGA recommendations—conferred protection against experimental colitis in pigs. Pigs in the full-FV group did not exhibit clinical signs of disease and maintained gut microbiome diversity, metagenome function, and an unaltered transcriptome in PC mucosa, despite taxonomic differences in the microbiome induced by DSS provocation. In contrast, pigs in the half-FV and DSS-positive groups developed diarrhea. However, mucosal healing occurred only in the half-FV group, which showed a differential transcriptome response characterized by enhanced expression of genes related to antioxidant activity, intestinal transport, mucosal healing and amino acid metabolism. Conversely, genes associated with host mucosal structure and detoxification were compromised in the DSS group. Similarly, microbiome diversity and metagenome function were altered only in the DSS group.

## Figures and Tables

**Table 1:** Differentially expressed genes (DEGs) in proximal colon mucosa of pigs after dextran sodium sulfate exposure.

		DSS-vs-Control		Half-DSS-vs_Control			Full-DSS-vs-DSS				
Gene ID	Variant #	FC	Adj *p*-Value	FC	Adj *p*-Value		FC	Adj *p*-Value	Gene Full Name	Function	Reported Effects, (PMID Reference)
*GSTA1*	*GSTA1_tv1*	*−4.4*	2.40 × 10^−3^						Glutathione S-transferase	Detoxification	Required for intestinal function, (PMID 2308931); detoxification (PMID 16819479), (PMID 16432375).
*GPR15*	*GPR15*	*−2.9*	1.00 × 10^−3^	−3.0	4.00 × 10^−4^				G Protein-Coupled Receptor 15	Colon transport	Chemoattractant receptor that facilitates colon homing of regulatory and effector T cells (PMID 33674764), (PMID 37457732).
*ENPEP*	*ENPEP_tv1*	*−2.7*	5.12 × 10^−2^						Glutamyl Aminopeptidase	Inflammation	Glutamyl aminopeptidase affected in mouse DSS colitis (PMID 29900035).
*CXCL10*	*CXCL10*						3.4	5.40 × 10^−3^	C-X-C Motif Chemokine Ligand 10	Immune response	CXC chemokine; CXCR3 ligand; cytokine; LPS-induced (PMID 17082649). Binding of this protein to CXCR3 results in pleiotropic effects, including stimulation of monocytes, natural killer cells, and T-cell migration, and modulation of adhesion molecule expression. CXCL10 is positively correlated with IBD (PMID 37228614).
*GZMA*	*GZMA*	*−2.5*	6.70 × 10^−3^						Granzyme A	Inflammation	Enhances gut inflammation (PMID 32640217).
*SCD*	*SCD*	*−2.4*	7.20 × 10^−3^	−2.3	1.00 × 10^−2^				Stearoyl-CoA Desaturase	Lipid metabolism	Biochemistry and physiology of lipid metabolism (PMID 19066317).
KLRD1L *	KLRD1L *	*−2.2*	8.78 × 10^−2^	−2.4	3.54 × 10^−2^				Killer Cell Lectin-Like Receptor Subfamily D, Member 1	Immune response	Killer cell lectin-like receptor subfamily D, member 1 (PMID 9486650).
*XCL1*	*XCL1*	*−2*	7.20 × 10^−3^						X-C Motif Chemokine Ligand 1	Intestinal immune function	XCR1 receptor ligand, facilitate intestinal XCR1(+) DC activation and migration, providing support for T-cell survival and function (PMID 27005831).
*GNLY*	*GNLY_tv2*	*−2*	2.84 × 10^−2^						Granulysin	Intestinal immune function	Antimicrobial peptide present in cytotoxic granules of cytotoxic T lymphocytes and natural killer cells; has antimicrobial activity (PMID 9756476).
*TARP*	*TARP_tv1*	*−2*	3.32 × 10^−2^						TCR Gamma Alternate Reading Frame Protein	Cancer related	Favorable prognosis in B-cell lymphoma (PMID 34178023).
*CBX3*	*CBX3_tv2*	*−1.9*	1.98 × 10^−2^						Chromobox 3	Cancer related	Tumor development (PMID 35573019).
*PSMA5*	*PSMA5_tv1*	*−1.5*	7.20 × 10^−3^						Proteasome 20S Subunit Alpha 5	Immune response	Antigen processing and presentation (PMID 24706783).
*PSMA4*	*PSMA4_tv1*	*−1.5*	1.51 × 10^−2^						Proteasome 20S Subunit Alpha 4	Immune response	Antigen processing and presentation (PMID 24706783).
*NAMPT*	*NAMPT*	*−1.5*	1.84 × 10^−2^						Nicotinamide Phosphoribosyl-transferase	Intestinal immune function	Activation of phagocytosis in inflammatory macrophages (PMID 35063804).
*POLR1F*	*TWISTNB*	*−1.4*	1.02 × 10^−2^						RNA Polymerase I Subunit F	Cancer related	Hub gene linked to T2D and cancer (PMID 35601016).
*PSMA2*	*PSMA2*	*−1.4*	2.62 × 10^−2^						Proteasome 20S Subunit Alpha 2	Immune response	Antigen processing and presentation (PMID 24706783).
*AP1S3*	*AP1S3_tv1*	*−1.4*	1.98 × 10^−2^						Adaptor-Related Protein Complex 1 Subunit Sigma 3	Cancer related	Oncogene in gastric cancer (PMID 37078294).
*TNS3*	*TNS3_tv1*	*1.3*	9.69 × 10^−2^	1.4	1.51 × 10^−2^				Tensin 3	Cancer related	Enhancement of tumorigenesis (PMID 33824309).
*ST6GALNAC2*	*ST6GALNAC2*	*1.4*	3.03 × 10^−2^						ST6 N-Acetylgalactosaminide Alpha-2,6-Sialyltransferase 2	Cancer related	ST6GALNAC2 mediates migration, adhesion, invasion, proliferation, and tumor angiogenesis in colorectal cancer cell lines (PMID 29030743).
*CPT1A*	*CPT1A_tv1*	*1.4*	3.50 × 10^−2^	1.6	1.50 × 10^−3^				Carnitine O-Palmitoyltransferase 1A	Cancer related	Lipid metabolism; long-chain fatty acid beta oxidation; found overexpressed in colon cancer (PMID 34213835); (PMID 32913185).
*TTC3*	*TTC3*	*1.5*	6.70 × 10^−3^				−1	3.92 × 10^−2^	Tetratricopeptide Repeat Domain 3	Protein quality control	Ubiquitin-protein transferase; inhibits cell proliferation (PMID 20059950).
*EGFR*	*EGFR_tv1*	*1.5*	6.60 × 10^−3^	1.4	4.32 × 10^−2^				Epidermal Growth Factor Receptor	Cancer related	Oncogene (PMID 29133145); oncogenic driver (PMID 37321664).
*PLXNA2*	*PLXNA2*	*1.7*	6.60 × 10^−3^	1.6	2.68 × 10^−2^				Plexin A2	Cancer related	Tumor-forming ability (PMID 36658114).
*ABCC5*	*ABCC5_tv1*	*1.7*	4.70 × 10^−2^						ATP-Binding Cassette Subfamily C Member 5	Intestinal transport	Turnover of intracellular cAMP at the basolateral membrane of columnar epithelial cells (PMID 22734885).
*SLC12A8*	*SLC12A8*	*1.7*	1.66 × 10^−2^						Solute Carrier Family 12 Member 8	Intestinal transport	Amino acid transporter (PMID 19472210).
*ANO1/TMEM16A*	*ANO1*	*1.8*	6.60 × 10^−3^						Anoctamin 1	Innate immune response	Positive regulator of epithelial mucus production (PMID 25770012); calcium-activated chloride channel critical for epithelial secretion (PMID 34221860).
*DDX59*	*DDX59_tv1*	*1.8*	1.10 × 10^−3^						DEAD-Box Helicase 59	Cancer related	DDX59 has an important role in cancer development (lung) through promoting DNA replication (PMID 28090355); prognostic biomarker with immune infiltrates (PMID 36081993).
*MUC2*	*MUC2*	*2.2*	2.00 × 10^−4^	1.8	2.21 × 10^−2^				Mucin 2, Oligomeric Mucus/Gel-Forming	innate immune response	Protects gastrointestinal barrier (PMID 35602503); intestinal mucin (PMID 24942678).
*SPDEF*	*SPDEF_tv1*	*2.2*	2.26 × 10^−2^						SAM Pointed Domain-Containing ETS Transcription Factor	Innate immune function	Positive regulator of Paneth cell differentiation (PMID 19549527); positive regulator of goblet cell differentiation (PMID 19786015).
*ADCY1*	*ADCY1_tv1*	*2.2*	6.00 × 10^−4^						Adenylate Cyclase 1	Cancer related	Colon cancer (PMID 33744851); resistance to treatment (PMID 31839819).
*MAML3*	*MAML3*	*2.7*	2.40 × 10^−3^	2.4	1.47 × 10^−2^				Mastermind-Like Transcriptional Coactivator 3	Cancer related	Tumorigenesis (PMID 33986121); malignancy (PMID 37351966).
*NKX2-2*	*NKX2-2_tv1*	*2.9*	1.66 × 10^−2^						NK2 Homeobox 2	Cancer related	Adenocarcinoma (PMID 36870059); novel transcriptional regulator of serine/glycine synthesis addiction across cancers (PMID 36932191).
*APOB*	*APOB*	*3.7*	1.61 × 10^−2^	4.3	5.80 × 10^−3^				Apolipoprotein B	Cancer related	Expressed in colitis-associated cancer; enriched in the PI3K-Akt pathway, stem cell pluripotency regulation, focal adhesion, Hippo signaling, and AMPK signaling pathways (PMID 35733095).
*H19*	*H19_tv1*	*4.2*	1.66 × 10^−2^	7.5	1.00 × 10^−4^				H19 Imprinted Maternally Expressed Transcript	Cancer related	Proliferation and metastasis (PMID 36038043); (PMID 33221454).
*TFF2*	*TFF2*	*4.6*	1.02 × 10^−2^	4.4	1.51 × 10^−2^				Trefoil Factor 2	Innate immune function	Intestinal epithelial repair (PMID 24696606); (PMID 37129719).
*FRMD1*	*FRMD1_tvX1*	*4.6*	3.32 × 10^−2^	8	5.00 × 10^−4^				FERM Domain-Containing 1	Cancer related	Dysregulated gene (PMID 34446027); (PMID 23076869).
*CLDN8*	*CLDN8*	*8*	4.60 × 10^−3^	6.7	1.45 × 10^−2^				Claudin 8	Cation barrier function	Negative regulator of paracellular Na+ transport (PMID 19000657); regulation of paracellular fluxes of amino acids (PMID 36986076); colitis (PMID 31526198).
*CA4*	*CA4*			3.3	7.40 × 10^−3^				Carbonic Anhydrase 4	Cancer related	Biomarker in colon adenocarcinoma (PMID 32031891); associated with better prognosis in cancer (PMID 37713974).
*ITGB4*	*ITGB4_tv3*			1.5	6.10 × 10^−3^				Integrin Subunit Beta 4	Immune response/cancer related	Integrin; ITGB4 can be a useful biomarker for colorectal cancer (PMID 31877793); favorable prognosis in oral cell carcinoma (PMID 37371594).
*EPPK1*	*EPPK1*			1.9	2.02 × 10^−2^				Epiplakin 1	Cancer related	Positive regulator of epithelial cell keratin intermediate filament formation (PMID 15671067); oncogenic role in proliferation signaling (PMID 37328487).
*CTSF*	*CTSF_tv1*			6.9	1.47 × 10^−2^				Cathepsin F	Immune response	Antigen processing and presentation (PMID 10748235); cathepsin anti-tumor effect (PMID 3492398).
*RABGAP1L*	*RABGAP1L_tv1*				−1.7	1.51 × 10^−2^			RAB GTPase-Activating Protein 1-Like	Cancer related	RABGAP1L knockdown associated with reduced protumor activity of colon cancer cells (PMID 36345155).
*ITGB5*	*ITGB5_tv1*			1.4	8.70 × 10^−3^				Integrin Subunit Beta 5	Cancer related	ITGB5 shows a close association with focal adhesion, ECM–receptor interaction, phagosome, and PI3K-Akt signaling pathway; upregulation associated with bad prognosis (PMID 35223869).
*NUGGC*	*NUGGC_tv1*				−2.5	3.47 × 10^−2^			Nuclear GTPase, Germinal Center-Associated	Cancer related	Reduction in NUGGC (SLIP-GC) levels in Burkitt lymphoma cell line Raji and in non-Hodgkin lymphoma cell lines resulted in increased DNA breaks and apoptosis (PMID 19734146).
*FRY*	*FRY_tvX7*			1.7	6.10 × 10^−3^				FRY Microtubule-Binding Protein	Cell cycle	Involved in the control of chromosome alignment, spindle organization, and Polo-like kinase-1 activation in mitosis (PMID 24403109).
*CYP2B6B* *	*CYP2B22*			6.2	4.01 × 10^−2^				Cytochrome P450 Family 1 Subfamily B Member 1	Drug metabolism	Drug metabolism; high homology to humans (PMID 36286316).
*PLEC*	*PLEC*			1.8	2.05 × 10^−2^				Plectin	Immune response, inflammation	Positive regulator of TLR4 signaling (PMID 21665277); maintains intestinal epithelial integrity and protects colon against colitis (PMID 33674761).
*TAX1BP3*	*TAX1BP3_tv1*				1.5	8.70 × 10^−3^			Tax-Binding Protein 3	Cancer related	PDZ domain-containing protein overexpressed in cancer (PMID 36892460); differential membrane protein in hepatocellular carcinoma (PMID 37740172).
*RAI14*	*RAI14*			1.7	1.56 × 10^−2^				Retinoic Acid-Induced 14	Cancer related	Retinoic acid-induced RAI14 is an important prognostic determinant for APC-mutant colon cancer patients (PMID 36934880).
*CANT1*	*CANT1_tv*			1.4	5.00 × 10^−2^				Calcium-Activated Nucleotidase 1	Cancer related	Upregulated and associated with poor prognosis in hepatocellular cancer (PMID 37858061); potential prognosis marker (PMID 38369265).
*SLC22A5*	*SLC22A5_tv2*				1.7	2.02 × 10^−2^			Solute Carrier Family 22 Member 5	Transporter	Organic cation transporter abrogates intestinal inflammation (PMID 19175620); upregulation associated with reduced colonic inflammation (PMID 32579962).
*SLC35E2B*	*SLC35E2B*			1.7	2.53 × 10^−2^				Solute Carrier Family 35 Member E2B	Disease	One report suggests it could represent a disease candidate gene associated with myopia (PMID 33711669).
*PLIN2*	*PLIN2*			1.8	1.51 × 10^−2^				Perilipin 2	Cancer related	Intimately related to ferroptosis in ulcerative colitis by machine learning studies (PMID 36923072); PLIN2 expressed in tumor-infiltrating immunocytes of oral cell carcinoma (PMID 35372038).
*MMP7*	*MMP7*			131	5.00 × 10^−4^				Matrix Metallopeptidase 7	Inflammation	Intestinal wound healing (PMID 15545168).
*LAMB3*	*LAMB3_tv1*			2	4.00 × 10^−4^				Laminin Subunit Beta 3	Cancer related	LAMB3 promotes intestinal inflammation in IBD (PMID 37454278).
*CYP1B1*	*CYP1B1*			4.5	2.02 × 10^−2^				Cytochrome P450 Family 1 Subfamily B Member 1	Inflammation	Upregulated in inflammation linked to IBD (PMID 36177008).
*MYH9*	*MYH9*			1.4	1.51 × 10^−2^				Myosin Heavy Chain 9	Cancer related	Poor prognosis related to colon cancer patients (PMID 36345155).
*ITGA3*	*ITGA3_tva*			1.4	1.51 × 10^−2^				Integrin Subunit Alpha 3	Cancer related	Identified in human colon cancer relapse (PMID 25096929).
*COL8A1*	*COL8A1_tv2*				4.3	4.32 × 10^−2^			Collagen Type VIII Alpha 1 Chain	Cancer related	Involved in progression and prognosis of colon adenocarcinoma (PMID 29497907).
*IL4I1L* *	*IL4I1L*			50.6	3.95 × 10^−2^				Interleukin 4 Induced 1	Amino acid metabolism	IL-4-induced; putative L-amino acid oxidase (PMID 29288206); controls essential amino acid depletion (PMID 37196768); immunomodulatory enzyme (PMID 17356132).
*LAMP1*	*LAMP1*			1.4	9.00 × 10^−3^				Lysosomal-Associated Membrane Protein 1	Antimicrobial response	Lysosomal membrane glycoprotein that plays an important role in lysosome biogenesis, lysosomal pH regulation, autophagy, and cholesterol homeostasis (PMID 37390818); autophagy genes linked with aberrant immune responses to pathogenic bacteria (PMID 31286804).
*KRT86*	*KRT86_tv1*			13.7	1.47 × 10^−2^				Keratin 86	Apoptosis	Differentially expressed gene in necroptosis (PMID 37403014).
*ARG1*	*ARG1_tv1*			10.1	8.70 × 10^−3^				Arginase 1	Inflammation	Arginase impedes the resolution of colitis by altering microbiome and metabolome (PMID 32721946); regulates pathogenesis of IBD (PMID 36040377).
*RBP4*	*RBP4*			21.8	3.95 × 10^−2^				Retinol-Binding Protein 4	Vit A metabolism, cancer related	Binding of RBP4 to NLRP3 promotes colonic epithelial cell pyroptosis in ulcerative colitis (UC) (PMID 36440907).
*ADAMTS16*	*ADAMTS16_tv1*				20.7	3.95 × 10^−2^			ADAM Metallopeptidase with Thrombospondin Type 1 Motif 16	Cancer related	Metalloprotease with tumor suppressor activity reported in certain epithelial cancers (PMID 30081852).
*SCGB2A2*	*SCGB2A2*			25.7	1.01 × 10^−2^				Secretoglobin Family 2A Member 2	Cancer related	Early marker for metastasis in breast cancer (PMID 32747636).
*TM4SF19*	*TM4SF19*			14.6	4.07 × 10^−2^				Transmembrane 4 L Six Family Member 19	Inflammation	Endothelial cell adherens junction and positive regulator of LPS signaling (PMID 33059922).
*ALDH3B1*	*ALDH3B1_tv1*				1.6	4.76 × 10^−2^			Aldehyde Dehydrogenase 3 Family Member B1	Antioxidant	Protective role of ALDH3B1 against oxidative stress (PMID 17382292).

* Superscript in a gene symbol indicates the gene is expressed only in swine. # When a specific variant of the gene was used to quantify expression. Green shades represent up-regulated genes, red shades represent down-regulated genes in treatment groups relative to negative control group.

**Table 2:** Differentially abundance analysis of taxonomic features.

C vs. DSS			C vs. Half-FV DSS			C vs. Full-FV DSS			DSS vs. Half-FV DSS			DSS vs. Full-FV DSS			Half-FV DSS vs. Full-FV DSS		
**Phylum**																	
	FC	FDR		FC	FDR		FC	FDR		FC	FDR		FC	FDR		FC	FDR
Ascomycota	2.4	2.33 × 10^−3^	Ascomycota	2.6	6.81 × 10^−4^	Ascomycota	3.9	3.59 × 10^−8^	Firmicutes	−2.2	7.29 × 10^−4^	Firmicutes	−1.9	1.02 × 10^−2^			
Firmicutes	2.0	1.25 × 10^−2^	Chlamydiae	14.0	1.79 × 10^−3^	Spirochaetes	0.3	5.49 × 10^−2^									
Euryarchaeota	4.4	1.47 × 10^−2^	Euryarchaeota	4.0	3.25 × 10^−2^												
**Class**																	
Saccharomycetes	3.6	9.86 × 10^−4^	Saccharomycetes	4.1	1.55 × 10^−5^	Saccharomycetes	6.3	9.38 × 10^−10^	Elusimicrobia	5.6	4.88 × 10^−3^	Bacilli	−2.9	4.43 × 10^−2^	Deltaproteobacteria	−2.3	2.85 × 10^−2^
Bacilli	3.7	7.40 × 10^−3^	Chlamydiia	14.5	2.86 × 10^−3^				Bacilli	−2.8	3.72 × 10^−2^						
Methanobacteria	5.4	3.13 × 10^−2^	Deltaproteobacteria	2.4	1.82 × 10^−2^				Deltaproteobacteria	2.1	3.72 × 10^−2^						
			Caldilineae	0.6	2.97 × 10^−2^												
			Methanobacteria	4.8	3.47 × 10^−2^												
**Order**																	
Saccharomycetales	3.6	1.20 × 10^−3^	Saccharomycetales	4.2	7.30 × 10^−5^	Saccharomycetales	6.4	9.38 × 10^−9^	Veillonellales	−3.7	1.11 × 10^−3^				Desulfovibrionales	−4.0	2.24 × 10^−3^
Bifidobacteriales	3.5	2.96 × 10^−3^	Desulfovibrionales	4.6	1.86 × 10^−3^				Elusimicrobiales	5.7	1.11 × 10^−3^						
Lactobacillales	4.3	9.09 × 10^−3^	Chlamydiales	22.2	1.86 × 10^−3^				Desulfovibrionales	3.5	4.29 × 10^−3^						
Methanobacteriales	5.5	2.92 × 10^−2^															
**Family**																	
Saccharomycetaceae	4.2	9.58 × 10^−4^	Saccharomycetaceae	4.9	5.64 × 10^−5^	Saccharomycetaceae	7.6	6.89 × 10^−9^	Veillonellaceae	−3.6	3.14 × 10^−3^				Desulfovibrionaceae	−4.1	1.38 × 10^−2^
Bifidobacteriaceae	3.5	6.69 × 10^−3^	Desulfovibrionaceae	4.7	2.94 × 10^−3^	Pichiaceae	3.3	1.35 × 10^−5^	Elusimicrobiaceae	5.7	8.11 × 10^−3^						
Lactobacillaceae	5.4	4.67 × 10^−2^	Chlamydiaceae	22.3	3.90 × 10^−3^	Debaryomycetaceae	2.9	6.09 × 10^−5^	Desulfovibrionaceae	3.7	1.93 × 10^−2^						
			Debaryomycetaceae	2.2	1.86 × 10^−2^	Trichomonascaceae	2.4	3.59 × 10^−4^									
			Pichiaceae	2.2	2.00 × 10^−2^	Phaffomycetaceae	3.0	8.55 × 10^−3^									
			Trichomonascaceae	2.0	2.35 × 10^−2^												

Negative binomial models (DESeq2) were used for differential abundance testing of taxonomic features. Differences were compared between groups. *p*-values were calculated with the LRT test. Significance was determined by FDR < 0.05.

## Data Availability

The metagenome and RNAseq data generated and analyzed in this manuscript have been deposited in the Gene Expression Omnibus (GEO) repository under accession number GSE317287/PRJNA1406675.

## Supplementary Materials

The following supporting information can be downloaded at: https://www.mdpi.com/article/10.3390/nu18060937/s1, Figure S1: Feed consumption; Figure S2. Supplementary Volcanos; Figure S3: Microbiome composition; Table S1. Swine diet composition; Table S2. Clinical Measures; Table S3. Morphometric Analysis PCM; Table S4. Differential genus abundance; Table S5. SEED functions; Table S6. KEGG and EC abundance.

## Abbreviations

The following abbreviations are used in this manuscript

DGA	Dietary Guidelines for Americans
DSS	Dextran sodium sulfate
DEG	Differentially expressed gene
FV	Fruits and vegetables
IBD	Inflammatory bowel disease
LRT	Likelihood-ratio test
NR	Non-redundant transcript library
PC	Proximal colon
PCM	PC mucosa
UC	Ulcerative colitis
FOB	Fecal occult blood
