# Supplementary figures and images for: Fruit and Vegetable Supplemented-Diet Ameliorates Dextran Sodium Sulfate (DSS)-Induced Colitis by Modulating Host Transcriptome and Gut Metagenome Response
Source: Nutrients. 2026 Mar 16;18(6):937. doi: 10.3390/nu18060937 (PMC13029192; doi:10.3390/nu18060937)

## Slide 1
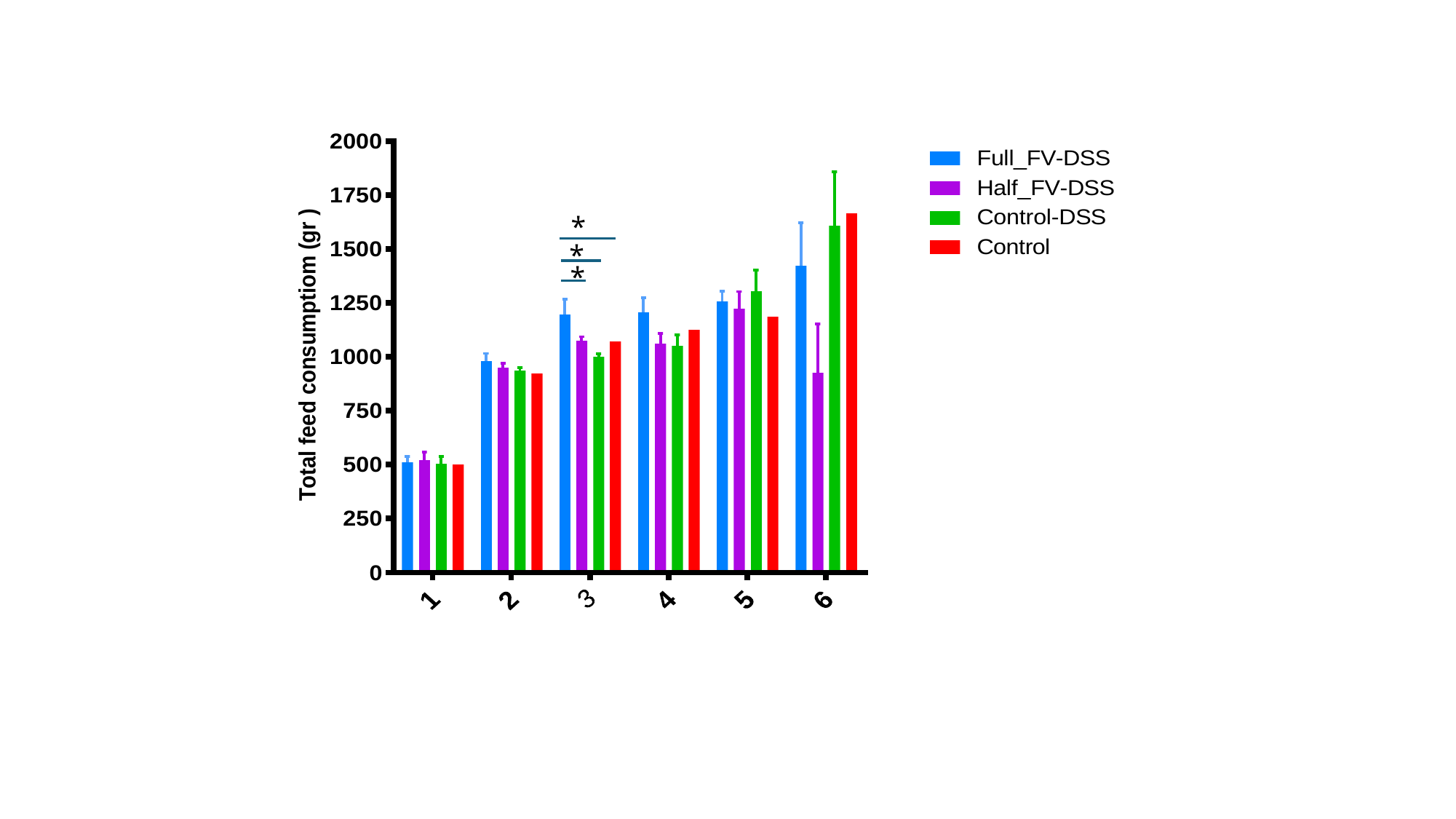

*
*
*

Supplement: Supplementary file 1 [file nutrients-18-00937-s001.zip › FigS1_Feed consumptiom.pptx]

## Slide 1
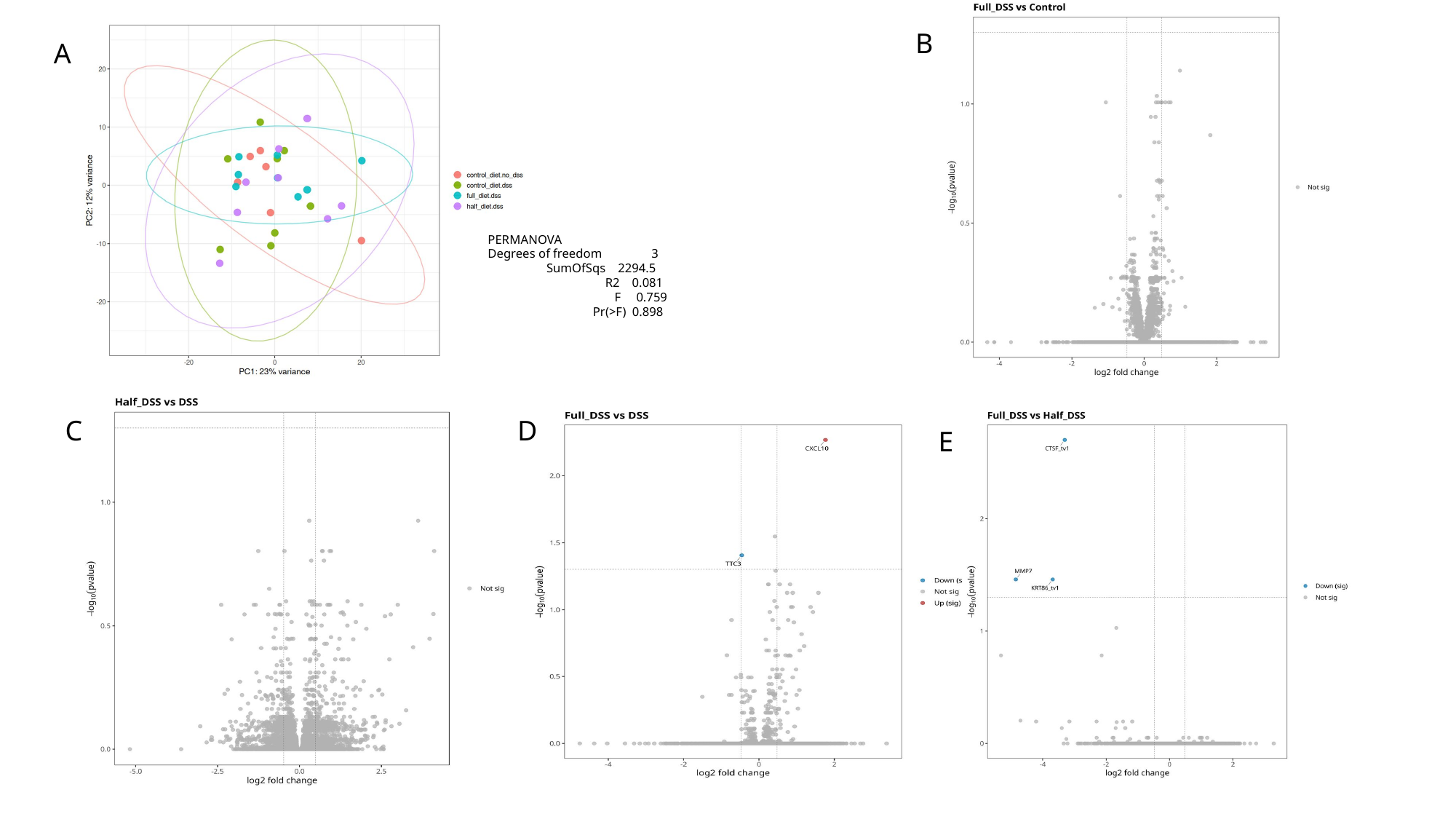

B
A
C
D
E
PERMANOVA
Degrees of freedom 3
 SumOfSqs 2294.5
 R2 0.081
 F 0.759
 Pr(>F) 0.898

Supplement: Supplementary file 1 [file nutrients-18-00937-s001.zip › FigS2_Supplemntary Volcanos.pptx]

## Slide 1
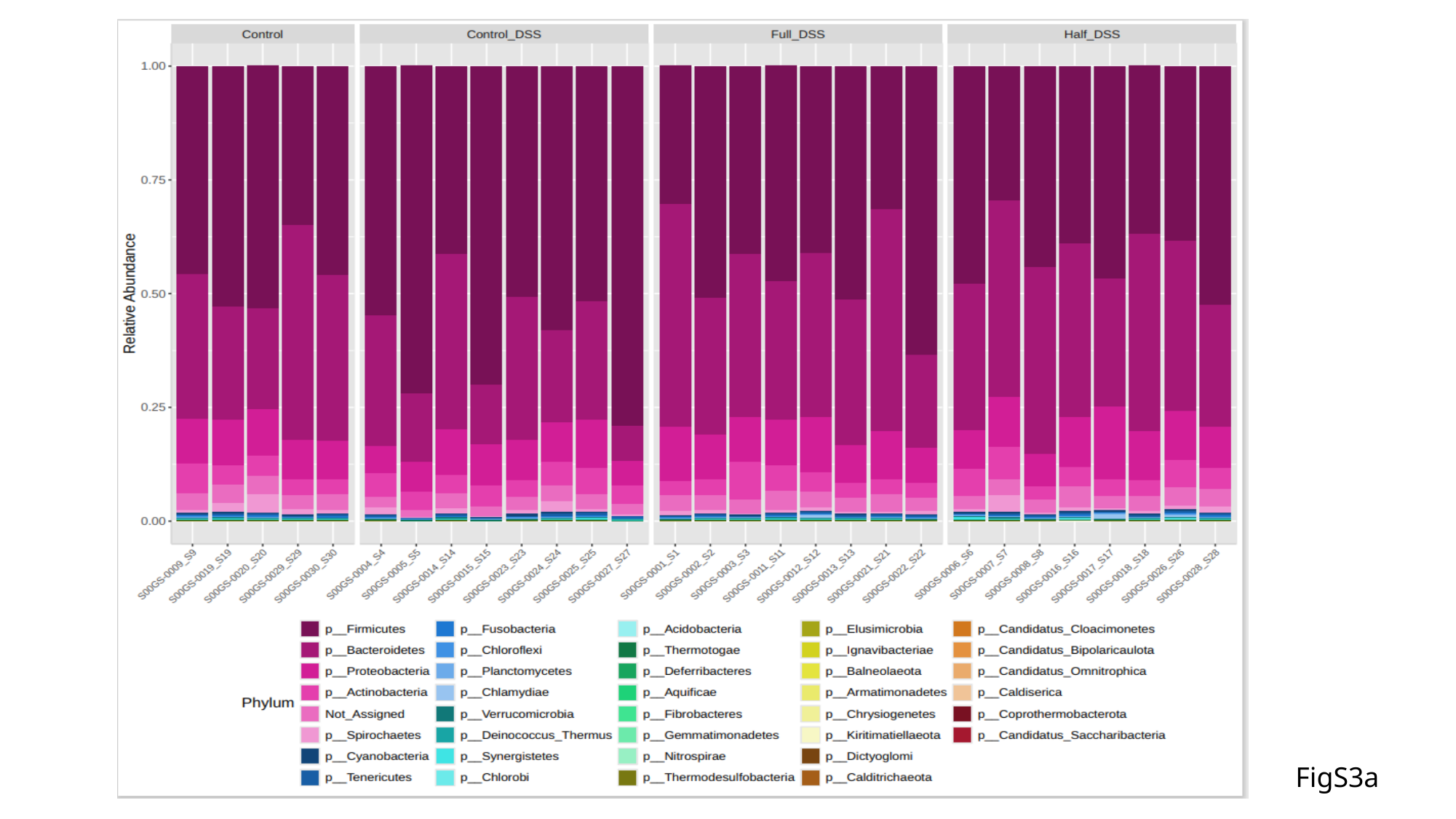

FigS3a

## Slide 2
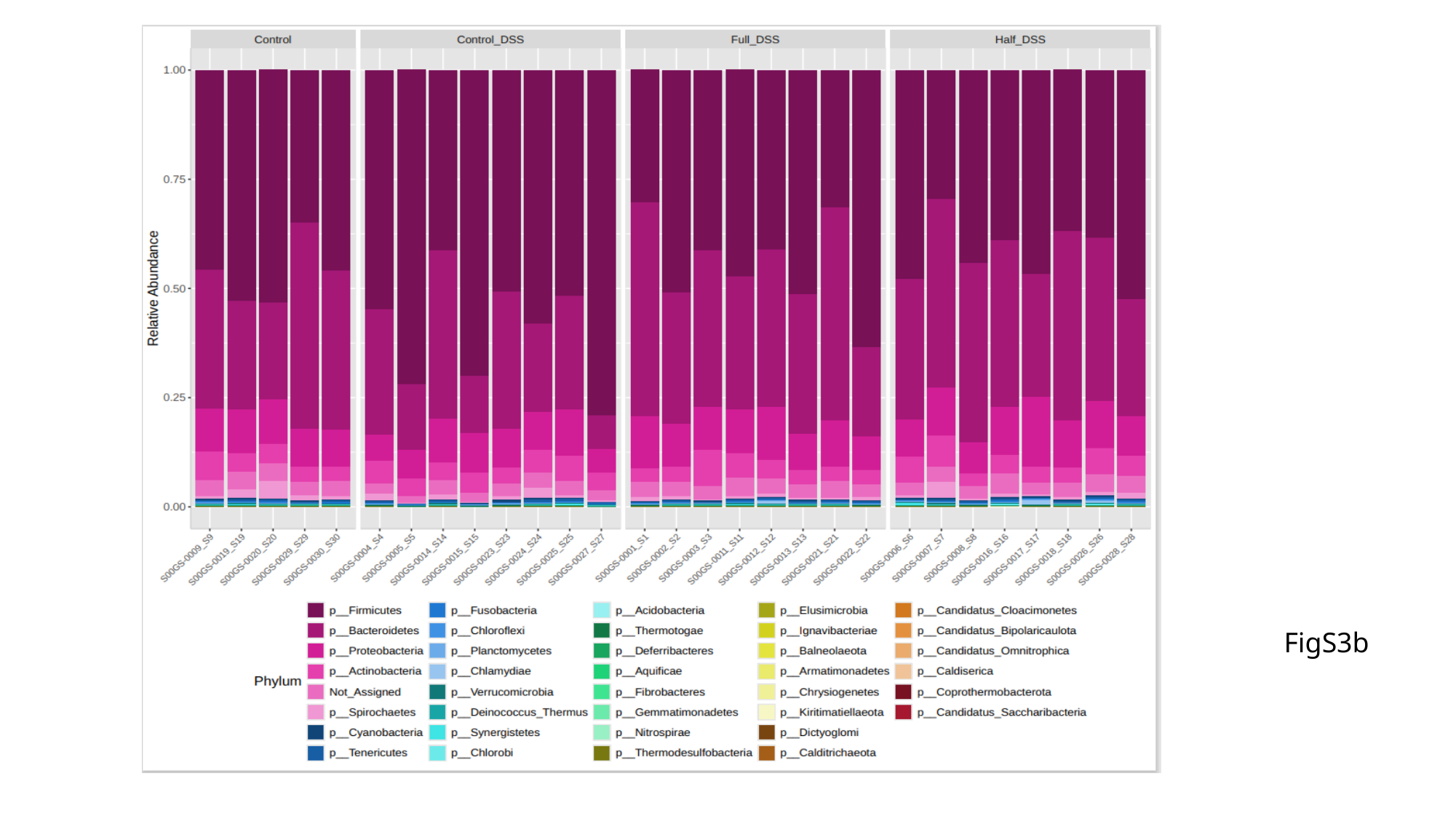

FigS3b

## Slide 3
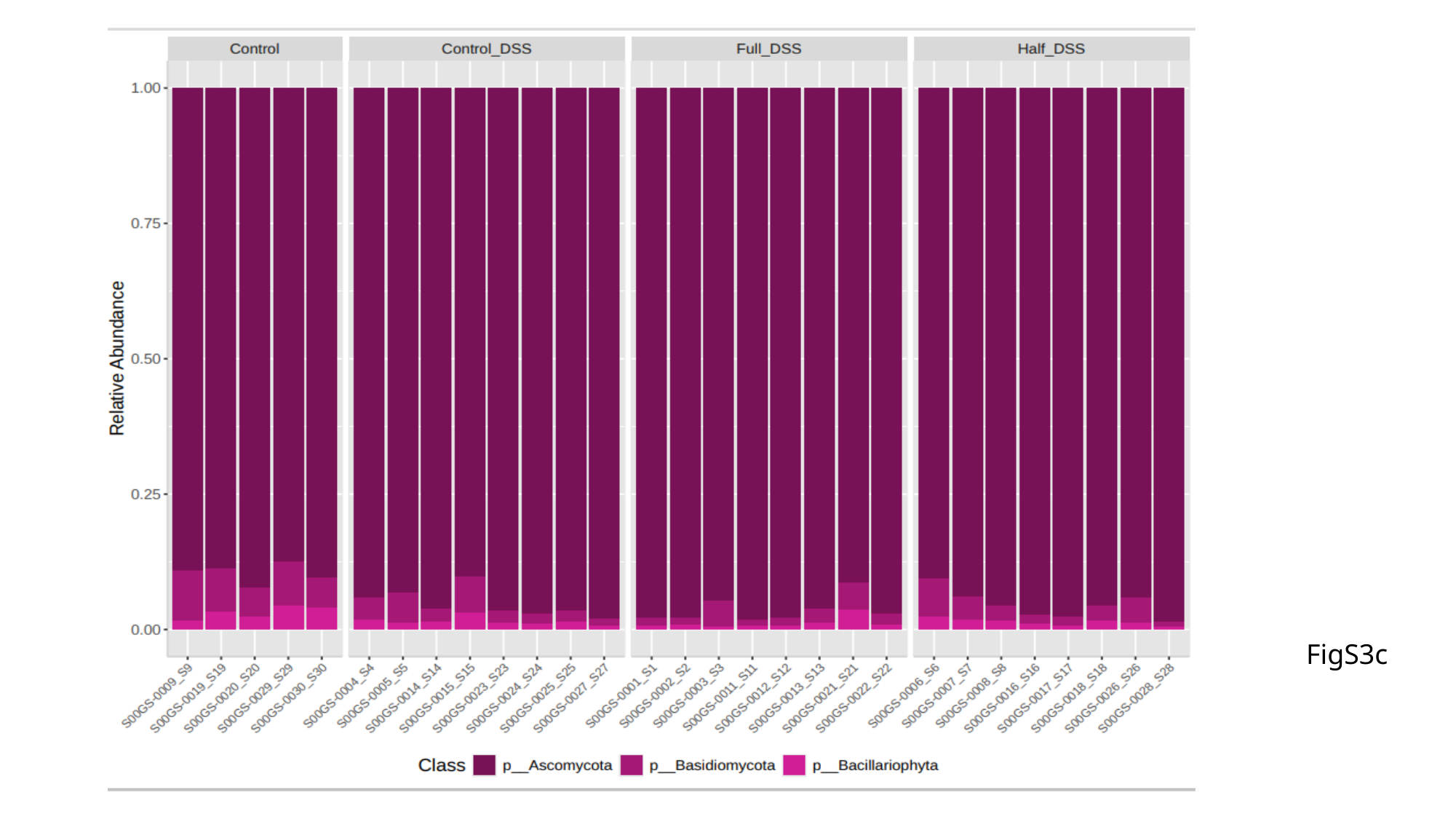

FigS3c

Supplement: Supplementary file 1 [file nutrients-18-00937-s001.zip › FigS3_Microbiome composition.pptx]
